# Effects of *HOX* family regulator-mediated modification patterns and immunity characteristics on tumor-associated cell type in endometrial cancer

**DOI:** 10.1186/s43556-024-00196-w

**Published:** 2024-08-14

**Authors:** JiaoLin Yang, JinPeng Li, SuFen Li, YuTong Yang, HuanCheng Su, HongRui Guo, Jing Lei, YaLin Wang, KaiTing Wen, Xia Li, SanYuan Zhang, Zhe Wang

**Affiliations:** 1 Department of Gynecology, First Hospital of Shanxi Medical University, Taiyuan, 030001 China; 2 Shanxi Medical University, Taiyuan, 030001 China

**Keywords:** *HOX* gene, Endometrial cancer, Tumor microenvironment, scRNA-seq, Cancer-associated fibroblasts (CAFs)

## Abstract

Endometrial cancer (UCEC) is one of three major malignant tumors in women. The *HOX* gene regulates tumor development. However, the potential roles of *HOX* in the expression mechanism of multiple cell types and in the development and progression of tumor microenvironment (TME) cell infiltration in UCEC remain unknown. In this study, we utilized both the The Cancer Genome Atlas (TCGA) database and International Cancer Genome Consortium (ICGC) database to analyze transcriptome data of 529 patients with UCEC based on 39 *HOX* genes, combing clinical information, we discovered *HOX* gene were a pivotal factor in the development and progression of UCEC and in the formation of TME diversity and complexity. Here, a new scoring system was developed to quantify individual *HOX* patterns in UCEC. Our study found that patients in the low *HOX* score group had abundant anti-tumor immune cell infiltration, good tumor differentiation, and better prognoses. In contrast, a high *HOX* score was associated with blockade of immune checkpoints, which enhances the response to immunotherapy. The Real-Time quantitative PCR (RT-qPCR) and Immunohistochemistry (IHC) exhibited a higher expression of the *HOX* gene in the tumor patients. We revealed that the significant upregulation of the *HOX* gene in the epithelial cells can activate signaling pathway associated with tumour invasion and metastasis through single-cell RNA sequencing (scRNA-seq), such as nucleotide metabolic proce and so on. Finally, a risk prognostic model established by the positive relationship between *HOX* scores and cancer-associated fibroblasts (CAFs) can predict the prognosis of individual patients by scRNA-seq and transcriptome data sets. In sum, *HOX* gene may serve as a potential biomarker for the diagnosis and prediction of UCEC and to develop more effective therapeutic strategies.

**Supplementary Information:**

The online version contains supplementary material available at 10.1186/s43556-024-00196-w.

## Introduction

UCEC, comprising a spectrum of malignant epithelial tumors originating from the uterine lining, was among the principal gynecological malignancies, constituting approximately 20% to 30% of all cancers within the female reproductive system. Notably, a study conducted in the United Kingdom documented a twofold increase in incidence between 1993 and 2014, with projections indicating a continued rise through 2035 [1]. The *HOX* genes, a family of highly conserved DNA sequences found across eukaryotes [2], played pivotal roles in developmental regulation, orchestrating not only growth and development but also the establishment of the immune system [3]. In humans, these genes are organized into four clusters—*HOXA*, *HOXB*, *HOXC*, and *HOXD*—residing on chromosomes 7, 17, 12, and 2, respectively. The transcription factors encoded by *HOX* genes exhibit dual functions, acting as both tumor suppressors and oncogenes [4]. MicroRNAs (miRNAs) embedded within the *HOX* loci can modulate *HOX* expression, impacting apoptosis and thus influencing carcinogenesis [5]. Research has identified specific members of the *HOX* family with tumor-suppressive capabilities; for instance, *HOXB13* has been characterized as a tumor suppressor [6–8]. Similarly, the ectopic expression of *HOXA 5* in lung adenocarcinoma cell lines has been shown to inhibit cell migration, invasion, and filopodia formation in vitro, while concurrently reducing metastatic potential in vivo [9]. Furthermore, a significant downregulation of *HOXD 10* mRNA expression was observed in gastric cancer tissues, correlating with enhanced tumor cell proliferation, migration, and invasiveness [10].

The tumor immune response was a sophisticated and dynamic interplay that exerts dual influences on the genesis and evolution of cancer. This intricate process unfolded in three distinct phases: elimination, equilibrium, and escape. In the initial phase of tumor development, immune effector cells, including T lymphocytes (TLs), mounted a defense against cancerous cells. TLs could produce inflammatory cytokines and differentiate into effector cytotoxic T lymphocytes (CTLs) capable of directly killing tumor cells [11, 12]. Furthermore, CTLs activation can modulate immune signaling within the TME, potentially enhancing antitumor immunity [13]. As the tumor advances, it adopted strategies to evade immune surveillance. Tumor cells secreted immunosuppressive cytokines such as interleukin-10 (IL-10), which dampened the activity of activated CTLs, facilitating tumor expansion and immune escape [14]. Despite this, research exploring the impact of *HOX* genes on immunity within the TME remained sparse. One study elucidated that *HOX* antisense transcript expression correlates with genes involved in immune cell infiltration, distinct immune subtypes, and the immunological response to cancer [15]. In the context of colorectal cancer, elevated expression of *HOXC 6* had been linked to the activation of cytokine pathways, upregulation of chemokines that attracted T cells, infiltration of immune cells, and the expression of immune checkpoint molecules [16]. Aberrant expression of specific *HOX* genes, notably *HOXA 10*, *HOXB 9*, and *HOXD 10*, had been observed in UCEC [17–20]. Dysregulated *HOX* gene expression activated several pathways, including PI3K/Akt, which fostered endometrial carcinoma cell proliferation and simultaneously hinder apoptosis [21].

CAFs represented a prominent cellular component within the TME. Activated CAFs exerted their influence by secreting a plethora of cytokines and chemokines, including CXC ligand 2 (CXCL2), which facilitated tumor cell proliferation and metastasis [22]. Recent investigations had unveiled the capacity of CAFs to induce angiogenesis in endometrial malignancies via IL-10 secretion, thereby accelerating tumor advancement [23]. Despite the growing understanding of CAFs' contributions to cancer, the specific impact of *HOX* genes on immunity, the modulation of the TME, and their interaction with CAFs remained largely uncharted territories. Whether *HOX* genes can potentiate the onset and progression of UCEC through these mechanisms was an open question that warrants further scrutiny.

In this study, aiming to develop a more effective and innovative methodology for evaluating the clinical relevance of UCEC and predicting immunotherapy efficacy and prognosis with greater precision, we integrated *HOX*-related genomic information from 529 endometrial cancer samples and evaluated the role of *HOX*-related genes in endometrial cancer, combined with associated immune and TME cell infiltration features. We also constructed a scoring system to quantify *HOX* expression patterns in individual patients and combined the scores with immune, pharmacotherapy, and related clinical traits to further guide clinical treatment. Moreover, we evaluated expression of related genes and immune cell infiltration on the prognosis of patients with UCEC. Finally, we developed a prognostic risk model to further assess patient outcomes. Our study provides a new approach to guide more effective clinical practice and therapy for patients with endometrial cancer.

## Results

### Identification of *HOX* family-related differential expression genes (DEGs) with prognostic significance and genomic variance analysis in the UCEC cohort

To investigate the clinical relevance of *HOX* gene family members in endometrial carcinoma, we examined the effects of 39 *HOX* genes pan-cancer based on 4 aspects: disease-free interval (DFI), disease-specific survival (DSS), overall survival (OS), and progression-free survival (PFS). The results showed that the expression of 28 different *HOX* genes was associated with significant differences in DFI, DSS, OS, and PFS in UCEC (Fig. S1a). There were differences in the regulation of tumor grade between different *HOX* genes (Fig. S1b). And *HOX* genes can activate or inhibit certain signaling pathways, such as, receptor tyrosine kinase, ras-mitogen-activated protein kinase, cellcycle and so on (Fig. S1c). These results indicated that the *HOX* gene family plays a crucial role in UCEC. There was the whole process of our study (Fig. S2).

**Fig. 1:**
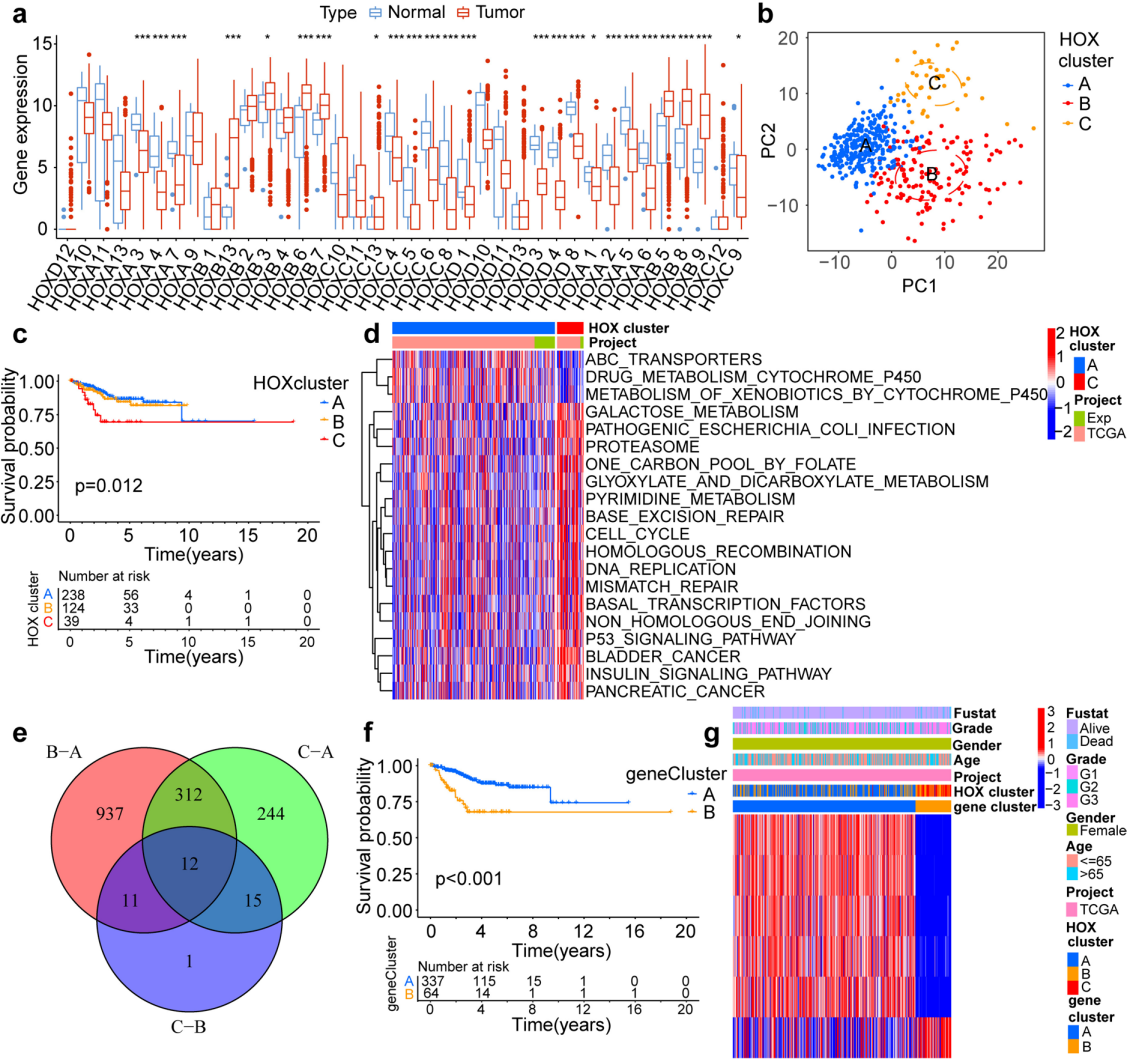
Differential expression of *HOX* genes. **a** Differences in the expression levels of *HOX* genes between normal and UCEC samples. The upper and lower ends of the boxes represent the interquartile range of values. The lines in the boxes represent the median value, whereas dots show the outliers. Asterisks represent the statistical *P*-value (*P < 0.05; **P < 0.01; ***P < 0.001). **b** Principal component analysis of the three patterns of *HOX* expression, showing a remarkable difference on gene profiles between different patterns. **c** Kaplan–Meier curves of overall survival (OS) for 575 patients with UCEC in the TCGA and ICGC cohorts with different *HOX* clusters. The numbers of patients in A, B, and C phenotypes were 238, 124, and 39, respectively (*P* = 0.012. Log-rank test). **d** GSVA enrichment analysis showing the state of metabolic pathways in distinct patterns between A and C clusters. The heatmap was used to visualize these biological processes. Red indicates activated pathways, whereas blue represents inhibited pathways. **e** 12 *HOX*-related DEGs between three *HOX* clusters are shown in the Venn diagram. **f** The survival curves of the *HOX* cluster-related gene signatures were estimated using the Kaplan–Meier plotter. (*P* < 0.001, Log-rank test). **g** Unsupervised clustering of overlapping *HOX* cluster-related DEGs for classifying patients into different genomic subtypes. Red indicates high expression, whereas blue represents low expression. Values represent the expression strength

To determine whether expression of *HOX* genes is involved in tumor formation, we studied its mutation frequency, copy number variation (CNV), and expression status. Among the 529 samples, 124 showed *HOX* gene mutations, with a frequency of 23.44%, including mostly missense, nonsense, and multiple hit mutations. *HOXA 1* and *HOXA 2* showed the highest mutation frequencies, follow by *HOXA 11, HOXC 10,* and *HOXC 6*. The only *HOX* gene without a mutation was *HOXB 4* (Fig. S3a). We further analyzed the CNV frequency in *HOX* gene, mostly consisting of copy number amplifications (Fig. S3b). The locations of CNV alterations in *HOX* genes on chromosomes were shown (Fig. S3c). *HOXA 1*–*7* are mostly concentrated on chromosome 7; *HOXB 1*–*3* are located on chromosome 17; *HOXC 6 and 10*–*13* are located on chromosome 12; and *HOXD 1, 3, 4,* and* 8*–*13* are located on chromosome 2, and they all presented as deletions. To explore the functional differences caused by CNV alterations in the *HOX* genes in UCEC, the Kyoto Encyclopedia of Genes and Genomes (KEGG) the Gene Ontology (GO) functional enrichment were performed. The results exhibited those genes were enriched in signaling pathways regulating pluripotency of stem cells, DNA-binding transcription activator activity, RNA polymerase II-specific and DNA-binding transcription activator activity (Figs. S3d and S3e). In addition, we compared the expression of the *HOX* gene between UCEC and normal tissues. There were obvious differences in the expression levels of *HOX* genes in the tumor and normal groups (Fig. 1a). These results suggest that the deletion status of *HOX* gene regulates its upregulation and downregulation in tumor tissues, and *HOX* gene plays a vital role in the occurrence and development of UCEC. Next, further analysis of the interactions between *HOX* genes and tumor formation revealed a strongly significant positive correlation between most of *HOX* genes, with the correlation coefficient between *HOXB 6* and *HOXB 5* being the highest (0.97) (Fig. S3f). These results indicate that *HOX* genes do not affect the development of tumors alone, but also have certain synergistic or antagonistic effects. In summary, this analysis showed the high heterogeneity of genetic and expression landscapes in *HOX* gene between normal and UCEC samples, suggesting that *HOX* gene expression imbalance plays a pivotal role in UCEC occurrence and progression.

### Identification of *HOX* phenotype-related DEGs and regulation patterns

To further ascertain the influence of *HOX* on UCEC, we obtained gene expression data and full clinical annotations for 401 UCEC patients. We used unsupervised clustering analysis and principal component analysis (PCA) to classify 401 patients with UCEC and identified three distinct modification patterns, including 238 cases of pattern A, 124 cases of pattern B, and 39 cases of pattern C (Figs. S4a and 1b). These clusters were named *HOX* clusters A, B, and C, respectively. Survival analyses indicated that these *HOX* clusters were significantly related to prognosis of patients with UCEC, with *HOX* cluster A exhibiting a prominent survival advantage (Fig. 1c).

Next, we examined characteristics of the three *HOX* clusters according to different clinical traits. We conducted unsupervised clustering of 39 *HOX* genes in the cohort with clinical annotations, including age, sex, tumor grade, and survival status. The heatmap not only revealed clinical characteristics of the three *HOX* clusters but also their correlation with *HOX* gene expression. There were significant differences in *HOX* transcriptional profiles among the three *HOX* clusters (Fig. S4b). *HOXB* sets exhibited a downward trend from *HOX* cluster A to B to C and showed notably low expression in *HOX* cluster C. We also found that the proportions of patients with higher stages and death outcomes were significantly higher in *HOX* cluster C.

Subsequently, we performed gene set variation analysis (GSVA) for the all DEGs to explore the functions of the three *HOX* clusters. As shown in the heatmap, between *HOX* clusters A and B, genes associated with DNA replication, cell cycle, RNA degradation, base excision repair, homologous recombination, mismatch repair, and nucleotide excision repair were more highly expressed in *HOX* cluster B than in *HOX* clusters A (Fig. S4c). There were some pathways with higher expression in *HOX* cluster C than in *HOX* cluster A, including those associated with DNA replication, cell cycle, P53 signaling, base excision repair, homologous recombination, and mismatch repair (Fig. 1d).

These results illustrate that aberrant expression of the *HOX* gene may affect cell biological behavior and cell signaling and DNA repair pathways, which are closely linked to tumor formation and development. These pathways include the cell cycle, DNA replication, P53 signaling, base excision repair, homologous recombination, mismatch repair, and nucleotide excision repair. These results were further supported by the fact that *HOX* gene expression was associated with tumor formation and progression.

### Construction of a novel risk score for regulation patterns mediated by *HOX* regulators in UCEC

**Fig. 2:**
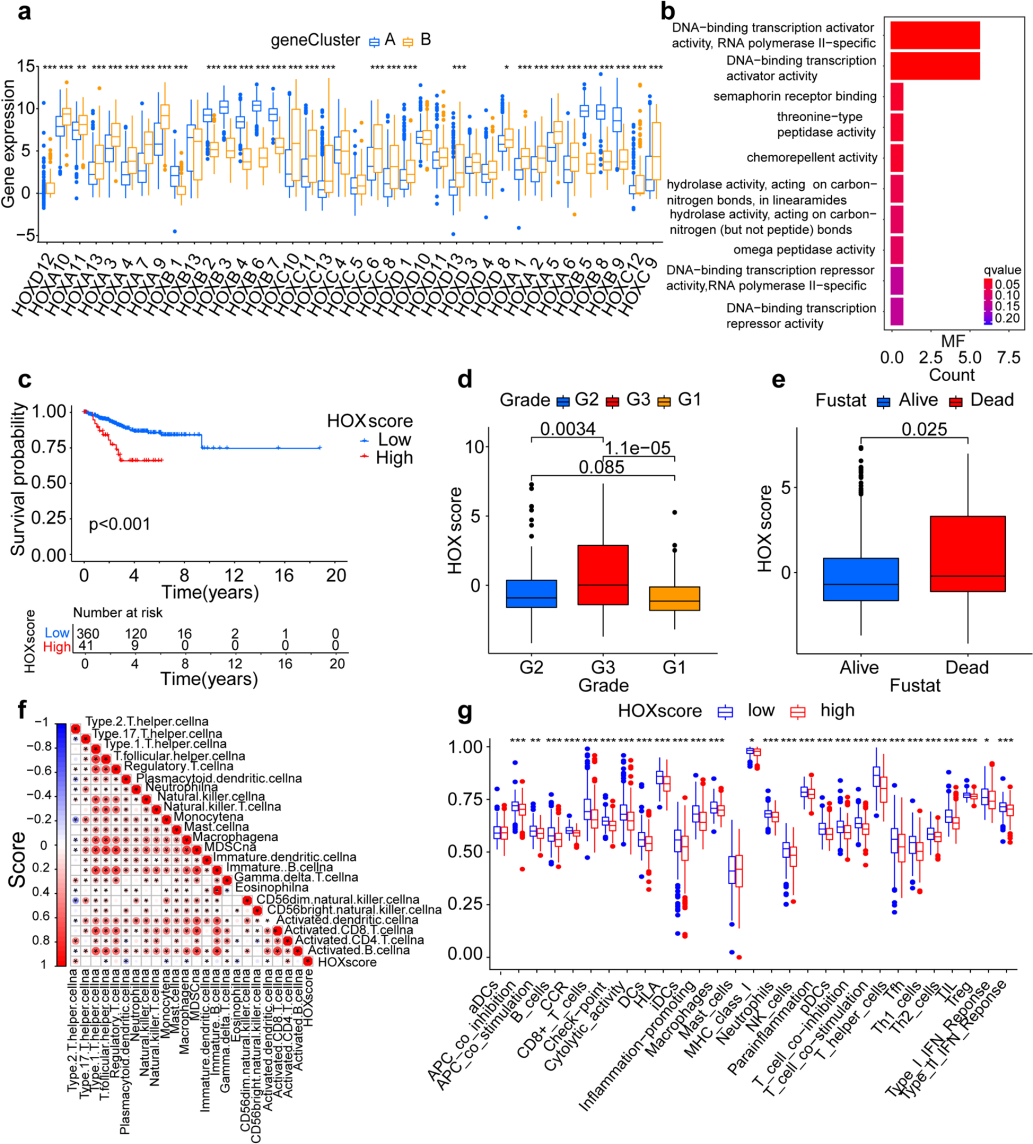
*HOX* scores combined with clinical features and immune cells. **a** Differences in the expression levels of *HOX* genes between two different genomic subtypes. The upper and lower ends of the boxes represent the interquartile range of values. The lines in the boxes represent the median value, whereas dots show the outliers. Asterisks represent the statistical *P*-value (*P < 0.05; **P < 0.01; ***P < 0.001). **b** GO functional enrichment analysis of 12 overlapping *HOX* cluster-related DEGs in molecular function (MF). Red indicates high expression, whereas blue represents low expression. The length of the bar chart indicates the count of enriched genes. **c** Survival analyses of low- (360 cases) and high- (41 cases) *HOX* score patient groups using Kaplan–Meier curves (*P* < 0.001, Log-rank test). **d** Differences in *HOX* score among distinct tumor grade groups. **e** Differences in *HOX* score among distinct survival state groups. **f** Correlations between *HOX* score and TME infiltrating cell types using Spearman analysis. Blue indicates negative correlation, whereas red represents positive correlation. Values represent the correlation strength. **g** Differences in the abundance of immune cells and pathways between low and high *HOX* score groups. The upper and lower ends of the boxes represent the inter-quartile range of values. The lines in the boxes represent the median value, whereas dots show the outliers. Asterisks represent the statistical *P* value (*P < 0.05; **P < 0.01; ***P < 0.001)

To investigate the potential biological behavior associated with each *HOX* gene pattern, we analyzed all of the DEGs in the three *HOX* clusters and determined 12 *HOX* cluster-related DEGs (Fig. 1e), including *HOXA 7*, *HOXA 9*, *HOXB 2*, *HOXB 3*, *HOXB 4*, *HOXB 5*, *HOXB 6*, *HOXB 7*, *HOXB 8*, *HOXC 4*, *SEMA3E* and *ASRGL1*, and then performed unsupervised clustering and PCA based on the these *HOX* cluster-related DEGs to classify patients into two different genomic subtypes named gene clusters A and B (Figs. S4d and S4e). Survival analysis indicated that gene clusters were significantly related to prognosis in patients with UCEC, and gene cluster A exhibited a prominent survival advantage (Fig. 1f). Next, we explored the characteristics based on different clinical traits using unsupervised clustering in the cohort. The heatmap revealed a significant difference in *HOX* cluster-related DEGs between the two gene clusters: most of the *HOX* cluster-related DEGs showed a low expression status in gene cluster B, and patients in *HOX* cluster C were mainly concentrated in gene cluster B (Fig. 1g). These results indicate that two distinct gene patterns are present in UCEC and are closely related to clinicopathological characteristics. Moreover, a prominent difference was noted in the expression of *HOX* between the two gene clusters, and *HOX* gene was the source of prominent differences in the two genomic phenotypes (Fig. 2a). To explore the characteristics of these *HOX* patterns in different clinical traits, we focused on the groups of tumor and normal. The unsupervised clustering by the DEGs revealed distinct patterns of *HOX* gene expression. To investigate the concrete regulating function of *HOX* gene in UCEC, further analysis was performed to elucidate the molecular mechanisms of the *HOX* gene and their interacting functions in three *HOX* clusters from RNA sequencing datasets (Fig. S5). *HOX* cluster A contained *HOXB 2* and *HOXB 3*. Compare to the normal samples, the major functions were downregulation in the tumor samples, including DNA-templated DNA replication, DNA replication, double-strand break repair, chromosome segregation, nuclear chromosome segregation, organelle fission. *HOX* cluster B contained *HOXB 6*, *HOXB 7* and *HOXB 13*; Major enriched functions included mitochondrial gene expression, mitochondrial translation, oxidative phosphorylation, aerobic respiration, proton motive force-driven ATP synthesis, purine nucleoside triphosphate biosynthetic process. These functions were downregulation in the tumor samples. *HOX* cluster C contained *HOXA 3*, *HOXA 4*, *HOXA 13*, *HOXC 4*, *HOXC 6* and *HOXD 13*. The major enriched functions included cell-substrate adhesion, small GTPase mediated signal transduction, cell–matrix adhesion, muscle organ development, regulation of supramolecular fiber organization, response to transforming growth factor beta. The functions above were upregulation in the tumor samples. To explore the relationship between biological behaviors and *HOX* gene expression in UCEC, we performed GO enrichment analyses of the 12 *HOX* cluster-related DEGs. GO enrichment analysis showed that *HOX*-related genes were enriched in multiple pathways, namely DNA-binding transcription repressor activity, DNA-binding transcription activator activity, RNA polymerase II-specific, and transcription regulator complex (Fig. 2b). These pathways play a regulatory role in the formation and development of tumors, further illustrating that *HOX*-related DEGs play a crucial role in the regulation of tumor cells.

As the above analyses were based on the entire cohort, they did not accurately predict the patterns of *HOX* expression in individual tumors. Therefore, we constructed a set of scoring models based on these cluster-related genes to quantify *HOX* gene expression in individual patients with UCEC and predict treatment response and prognosis of patients with UCEC. We termed this the *HOX* score. Based on the correlation of the *HOX* score with different *HOX* patterns and gene phenotypes, we determined the optimal cutoff value 3.6953 by “survminer” package, and divided 401 patients with UCEC into high (n = 41) and low (n = 360) *HOX* score groups. Next, we conducted survival analyses to assess the value of the *HOX* score in predicting patient outcomes. Kaplan–Meier curves showed that patients with low *HOX* scores had significantly better survival than those with high *HOX* scores (Fig. 2c). In this cohort, *HOX* cluster A and *HOX* gene cluster A were associated with lower *HOX* scores and better prognosis, verifying the robust and independent prognostic value of *HOX* gene signatures.

### Immune landscape was significantly associated with the expression of *HOX* score

To better validate the assessment potential of the *HOX* score in patients with UCEC, we combined this score with associated clinical features including age, tumor grade, and survival. We observed the proportions of patients with different clinical shapes in the high- and low-scoring groups. The histogram shows that in the high group, the proportion of patients with tumor grade 3 as well as dead was almost twice that of the low group (Figs. S6a-b); The proportion was similar in age between the different groups (Fig. S6c). In addition, box plots more intuitively showed the difference in *HOX* scores between the different clinical shape groups; patients in the G3 group had significantly higher *HOX* scores than those in the G1 and G2 groups (Fig. 2d); the scores were also higher in the death group than in the survival group (Fig. 2e). There were no statistical differences in *HOX* scores between the different age groups (Fig. S6d). The changes in the attributes of individual patients with UCEC were visualized using an alluvial diagram (Figs. S6e-g), and a close association was observed between *HOX* cluster C, gene cluster B, high *HOX* scores, tumor grade 3, and death. These results demonstrate that the *HOX* score can predict individual characteristics and has the potential to serve as a biomarker for assessing clinical characteristics and predicting prognosis in patients with UCEC.

To investigate the role of *HOX* in immune cell infiltration in the TME, TME cell-infiltrating characteristics were compared among the three *HOX* clusters. Notably, *HOX* cluster A was remarkably rich in innate immune cells, including activated macrophages, monocytes and plasmacytoid dendritic cell (Fig. S7a). Next, we investigated the relationship between *HOX* scores and different infiltrating immune cell characteristics. The *HOX* score was negatively correlated with numbers of activated dendritic cells, eosinophils, macrophages, monocytes, plasmacytoid dendritic cells, and T follicular helper cells, and positively correlated with activated CD 4 cells and type 2 T helper cells (Fig. 2f). Next, we compared the immune cell and immune function scores between high and low *HOX* score groups and noted remarkable differences. The low *HOX* score group generally had higher expression of immune cells, including B cells, CD8^+^ T cells, cytolytic activity, human leukocyte antigen (HLA), immature dendritic cells (iDCs), inflammation-promoting cells, natural killer (NK) cells, neutrophils, macrophages, T cell co-stimulation, T helper cells, T helper 1/T helper 2 cells, and type I/II interferon (IFN) responses (Fig. 2g). The heatmap showed that the expression of immune-infiltrating cells was lower in the high-score group than in the low-score group (Fig. S7b). Finally, we further investigated the relationship between immune scores and immune-infiltrating cells, revealing that the following cells had a positive correlation with immune scores: M1/M2 macrophages, NK resting cells, regulatory T cells, activated CD 4 memory T cells, and CD8^+^ T cells. Several other cells, such as naive B cells, plasma cells, activated dendritic cells, and eosinophils, were negatively correlated with immune scores (Fig. S7c).

Some soluble cytokine encoded by the *IFNG* gene are involved in tumor clearance, dormancy, escape, and progression [24, 25]. To explore the relationship between the *IFNG* gene and *HOX* scores, we examined the expression of the *IFNG* gene, which was found to be higher in the low *HOX* score group than in the high *HOX* score group (Fig. S7d). Myeloid-derived suppressor cells (MDSCs) are one of the main suppressive cell populations in the immune system. Some immature myeloid cells become MDSCs and exert immunosuppressive effects on CD8^+^ T cells [26]. To understand the differences in MDSCs and CD8^+^ T cells among the different groups, we observed their expression in the high- and low-scoring groups (Figs. S7e-f). Expression of MDSCs were lower in the low *HOX* score group than in the high *HOX* score group, whereas CD8^+^ T cell expression in low *HOX* score grouping was higher, illustrating that immune cell infiltration was more abundant in the low-score grouping. These results indicate that *HOX* scores reflect inhibition of immune cell infiltration in TMEs, leading to proliferation and metastasis of tumor cells.

### The role of *HOX* score in predicting tumor mutations and exacerbates tumor microsatellite instability

**Fig. 3:**
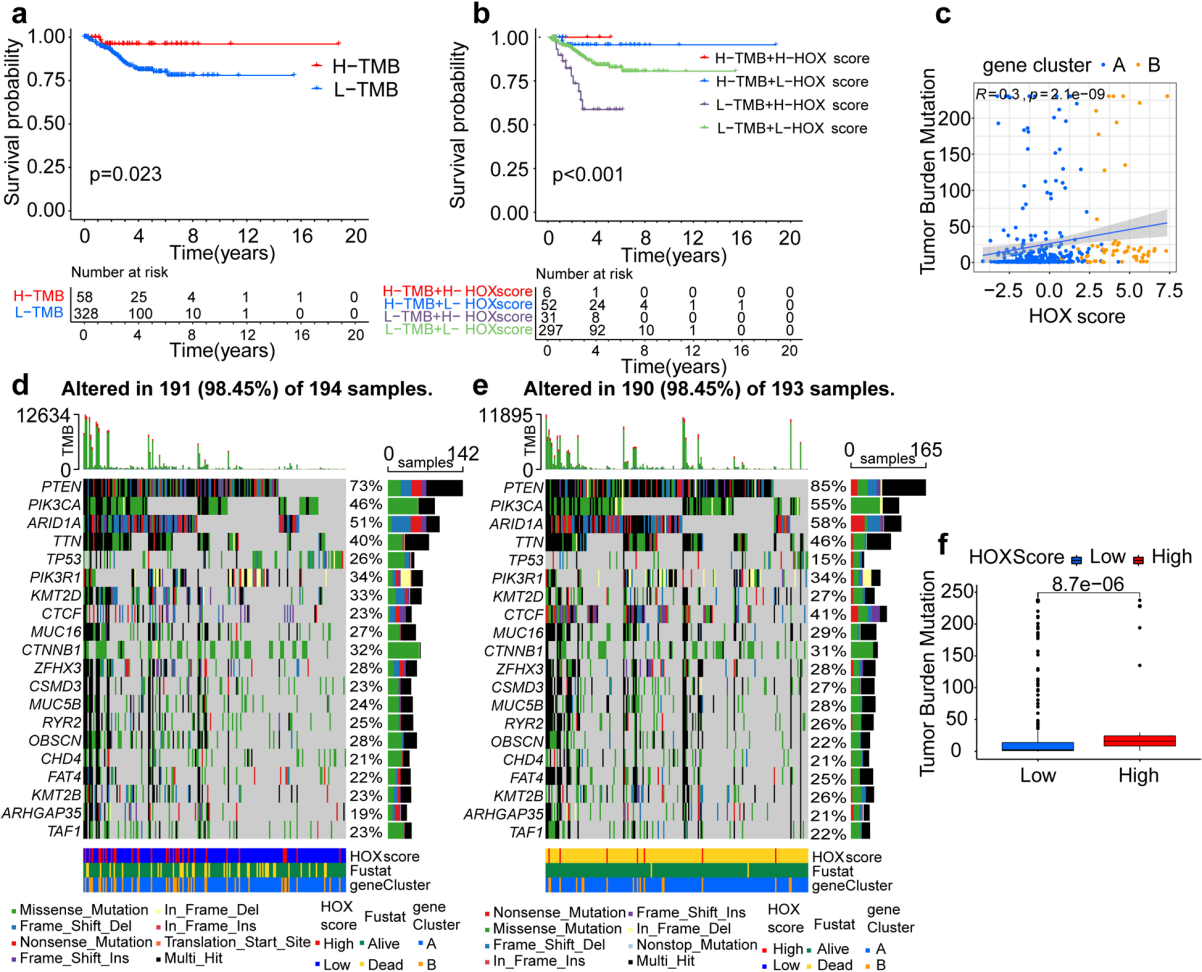
Association of *HOX* genes with TMB. **a** Kaplan–Meier curves of overall survival (OS) of patients with two TMB clusters. The numbers of patients in low and high TMB groups were 328 and 58, respectively (*P* = 0.023, Log-rank test). **b** Survival analyses of patients with both *HOX* score and TMB using Kaplan–Meier curves. H, high; L, Low; TMB (tumor mutation burden) (*P* < 0.001, Log-rank test). **c** The correlation between TMB and the *HOX* score in two gene clusters.** d-e** The waterfall plot of genetic alterations in patients with low (**d**) and high (**e**) *HOX* score. Each column represents individual patients. The upper barplot shows the TMB, while the number on the right indicates the mutation frequency in each gene. The right barplot shows the proportion of each variant type. **f** Differences in the TMB between low and high *HOX* score group

To understand the mutation status of the *HOX* gene in tumors, determine the effect of microsatellite instability, and predict the immunotherapy effect for patients in different *HOX* score groups, we divided the patients into two categories: L-TMB and H-TMB. Kaplan–Meier curves revealed that H-TMB patients had longer overall survival (Fig. 3a). Next, we evaluated overall survival among patients with UCEC combined with tumor mutation burden (TMB) and *HOX* scores. We observed that patients with both a high *HOX* score and high TMB showed a significant survival advantage (Fig. 3b). To explore the relationship between *HOX* scores and TMB, we performed a correlation analysis, which revealed a positive correlation between the two measures (Fig. 3c). We then analyzed somatic mutations between low and high *HOX* score groups in TCGA database and ICGC database cohorts using the “maftools” package and found higher mutation rates in genes associated with TMB in the high *HOX* score group (Figs. 3d-e). A box plot shows that the TMB was higher in the high-score group than in the low-score group (Fig. 3f). These results indicate that *HOX* promotes gene mutations in tumor cells. Next, we investigated the relationship between *HOX* score and tumor microsatellite instability (MSI). The proportion of patients with different MSIs in the low and high *HOX* score groups was analyzed, and we found that the proportion of MSI-H in the high *HOX* score group was nearly twice that in the low *HOX* score group (Fig. S7g). The *HOX* score was higher in the MSI group than in the microsatellite stability (MSS) group (Fig. S7h). These results indicate that factors related to high *HOX* scores may exacerbate tumor MSI. In addition, induced pluripotent stem cells can play a role in cancer therapy by inducing the proliferation of different immune cells [27]; therefore, we explored stem cell differences between the different *HOX* score groupings and found that the expression of immune cell proportion score (IPS) did not differ between them (Figs. S8a-d).

The above results show that *HOX* can promote gene mutations in tumor cells and exacerbate tumor MSI; therefore, patients in high *HOX* score groups appear to be better able to produce neoantigens, which are sensitive to immunotherapy.

### Exploration of effect on immune checkpoints and the association with *HOX* scores

**Fig. 4:**
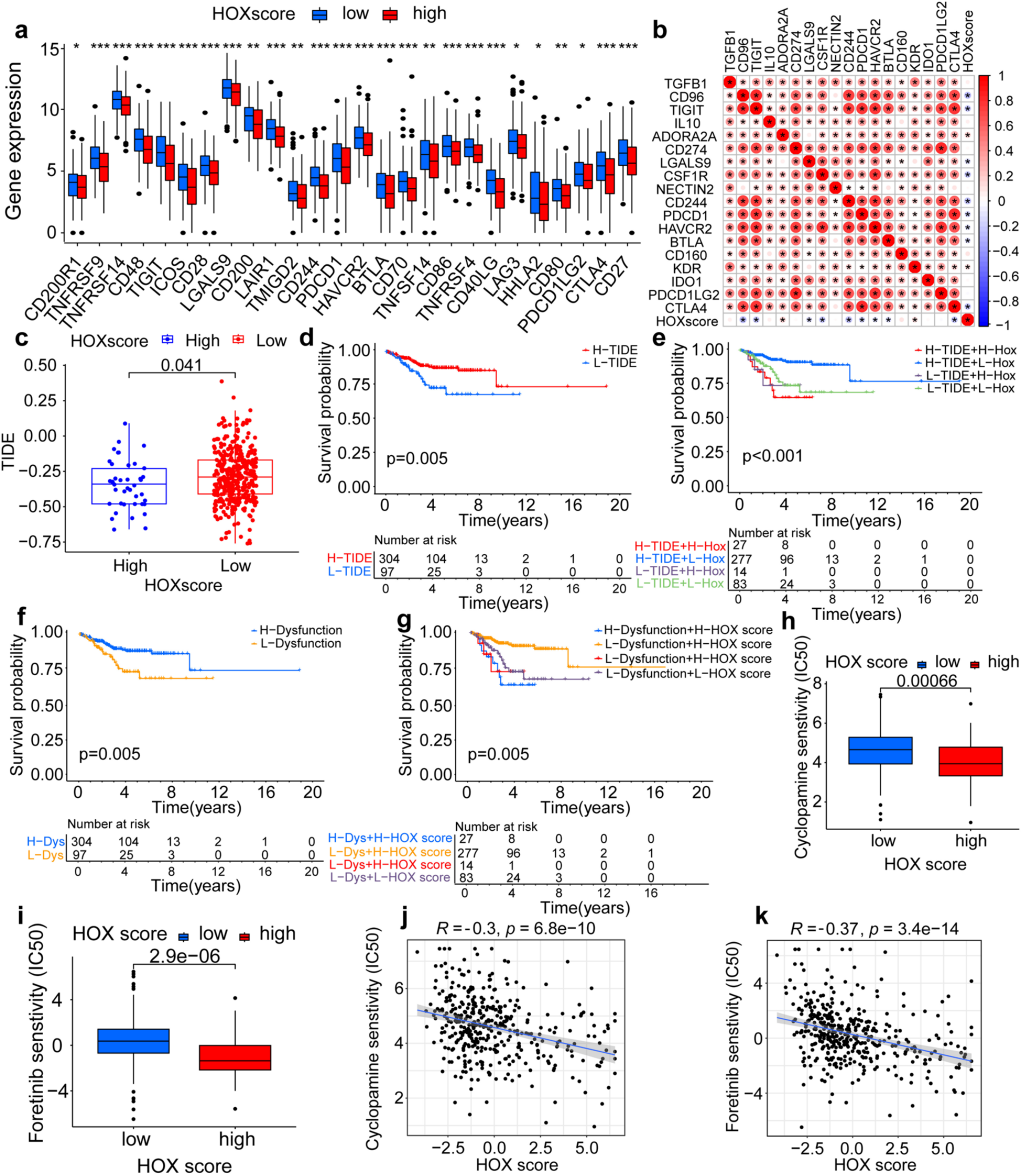
Combination of *HOX* genes with immune checkpoints and anti-tumor drugs. **a** Differences in the abundance of immune checkpoints between low and high *HOX* score groups. The upper and lower ends of the boxes represent the interquartile range of values. The lines in the boxes represent the median value, whereas black dots show the outliers. Asterisks represent the statistical *P* value (*P < 0.05; **P < 0.01; ***P < 0.001). **b** Correlations between *HOX* score and immune checkpoints using Spearman analysis. Blue indicates negative correlation, whereas red represents positive correlation. Values represent the correlation strength. **c** Comparison of the relative distribution of immune cells including TIDE, between high and low *HOX* score groups. **d** Kaplan–Meier curves of overall survival (OS) of patients. The numbers of patients in low- and high-TIDE groups were 97 and 304, respectively (*P* = 0.005, Log-rank test). **e** Survival analyses of patients with both *HOX* score and TIDE using Kaplan–Meier curves. (*P* < 0.001, Log-rank test). **f** Kaplan–Meier curves of overall survival (OS) of patients. The numbers of patients in the low- and high-immune dysfunction groups were 97 and 304, respectively (*P* = 0.005, Log-rank test). **g** Survival analyses of patients with both *HOX* score and immune dysfunction using Kaplan–Meier curves. (*P* < 0.001, Log-rank test). **h-i** Differences in the IC_50_ differences of anti-tumor drugs between different *HOX* score groups. **h**: cyclopamine. **i**: foretinib. **j-k** Correlation between anti-tumor drugs and *HOX* scores. **j**: cyclopamine. **k**: foretinib

We next explored the relationship between immune checkpoints and high and low *HOX* score groups. The high *HOX* score group presented a lower expression state for most immune checkpoints such as CD44, CD27, CD86, CTLA4, TIGIT and so on (Fig. 4a). Spearman’s analysis showed that checkpoints with the strongest correlation with *HOX* scores were CD244, CD96, and TIGIT; the correlation was negative (Fig. 4b). Next, we further verified the relationship between the three immune checkpoint genes and *HOX* scores (Figs. S8e-g). In addition, we verified the relationship between other checkpoint genes and *HOX* scores, and found that several checkpoints, namely CSF1R, BTLA, CTLA4, HAVCR2 and PDCD1, were negatively correlated with *HOX* scores (Figs. S8h-l). The results showed that a high *HOX* score was associated with a block in immune checkpoints, improved immune activation in patients with tumors, and enhanced immune response to immunosuppressants.

Subsequently, we performed GSVA enrichment analysis to compare differences in the activation states of immune cells and function between patients in distinct *HOX* score groups. As shown in the heatmap, the high *HOX* score group showed suppression of various immune cells and pathways such as CD8^+^ T cells, NK cells, T helper cells, and type I/II IFN responses (Fig. S9a).

To explore the correlation between *HOX* scores and the proportion of immune and stromal cells in TMEs and further examine differences in survival between the two *HOX* score groups, we analyzed Estimation of STromal and Immune cells in MAlignant Tumour tissues using Expression data (ESTIMATE) scores, immune scores, stromal scores, and tumor purity (Figs. S9b-e). The Violin plot shows that ESTIMATE, immune, and stromal scores were higher in the low-score group than in the high-score group, whereas tumor purity was lower in the low-score group. Therefore, compared with UCEC patients with a high *HOX* score, those with a low *HOX* score had tumors with more abundant immune and stromal components, and therefore had stronger immune function and better prognosis.

The use of immune checkpoint inhibitors (ICI) such as programmed death 1 (PD-1) and cytotoxic T lymphocyte-associated antigen-4 (CTLA-4) has become one of the most promising approaches in the field of cancer therapy. To verify how the *HOX* score relates to responses to anticancer drugs, we used Tumor Immune Dysfunction and Exclusion (TIDE) to predict the therapeutic effect of ICI based on pretreatment tumor profiles [28]. The TIDE score was significantly higher in the low *HOX* score group than in the high *HOX* score group, indicating that tumors in patients in the low *HOX* score group were more likely to experience immune escape and show less therapeutic effects in response to ICI (Fig. 4c). Consistent with this result, TIDE scores were also associated with prognosis. Patients with a high TIDE score had a distinctly better prognosis than those with low TIDE scores (Fig. 4d). We then determined overall survival among UCEC patients combined with TIDE and *HOX* scores, and found that patients with a combination of high TIDE and low *HOX* score exhibited a significant prognostic advantage; Patients with a combination of low TIDE and high *HOX* score had worst prognosis (Fig. 4e). We further analyzed targeted immune dysfunction. Kaplan–Meier curves revealed longer overall survival in the H-dysfunction + *HOX* score group (Fig. 4f). Survival analysis combining immune dysfunction with *HOX* scores showed the highest overall survival in the H-dysfunction and low *HOX* score groups, and lowest overall survival in the L-dysfunction and high *HOX* score groups (Fig. 4g). Therefore, regardless of the TIDE and immune dysfunction scores, patients in the low *HOX* score group consistently had better survival than those in the high *HOX* score group, indicating the value of the *HOX* score in predicting the therapeutic effect of ICI.

### *HOX* score can be used as a predictor of the therapeutic effect of anti-tumor drugs

Anti-tumor drugs are the main treatment for advanced tumors and after surgery. They have a killing effect on cells in multiple division stages (including the G0 stage), damaging DNA [29] or inhibiting enzymes that facilitate DNA replication [30]. To explore the differences in the treatment effects of anti-tumor agents between the different *HOX* score groups, we first compared the half maximal inhibitory concentration (IC_50_) differences of four anti-tumor drugs, namely cyclopamine, foretinib, fedratinib and pazopanib, between different *HOX* score groups; we found that the IC_50_ was higher in the low-score group than in the high-score group (Figs. 4h-i and S9f-g). Further, the correlation between IC_50_ values and *HOX* scores was negative (Figs. 4j-k and S9h-i). Next, we compared IC_50_ values of several other drugs, including rapamycin, doxorubicin, thapsigargin and nilotinib, with *HOX* scores. The IC_50_ values of drugs in the low *HOX*-score group were higher than of those in the high *HOX*-score group (Figs. S9j-l and S10a). These results indicate that patients with UCEC with a high *HOX* score have a better therapeutic response to anti-tumor drugs.

### The cell-type specific regulation of *HOX* gene in epithelial cell of UCEC

**Fig. 5:**
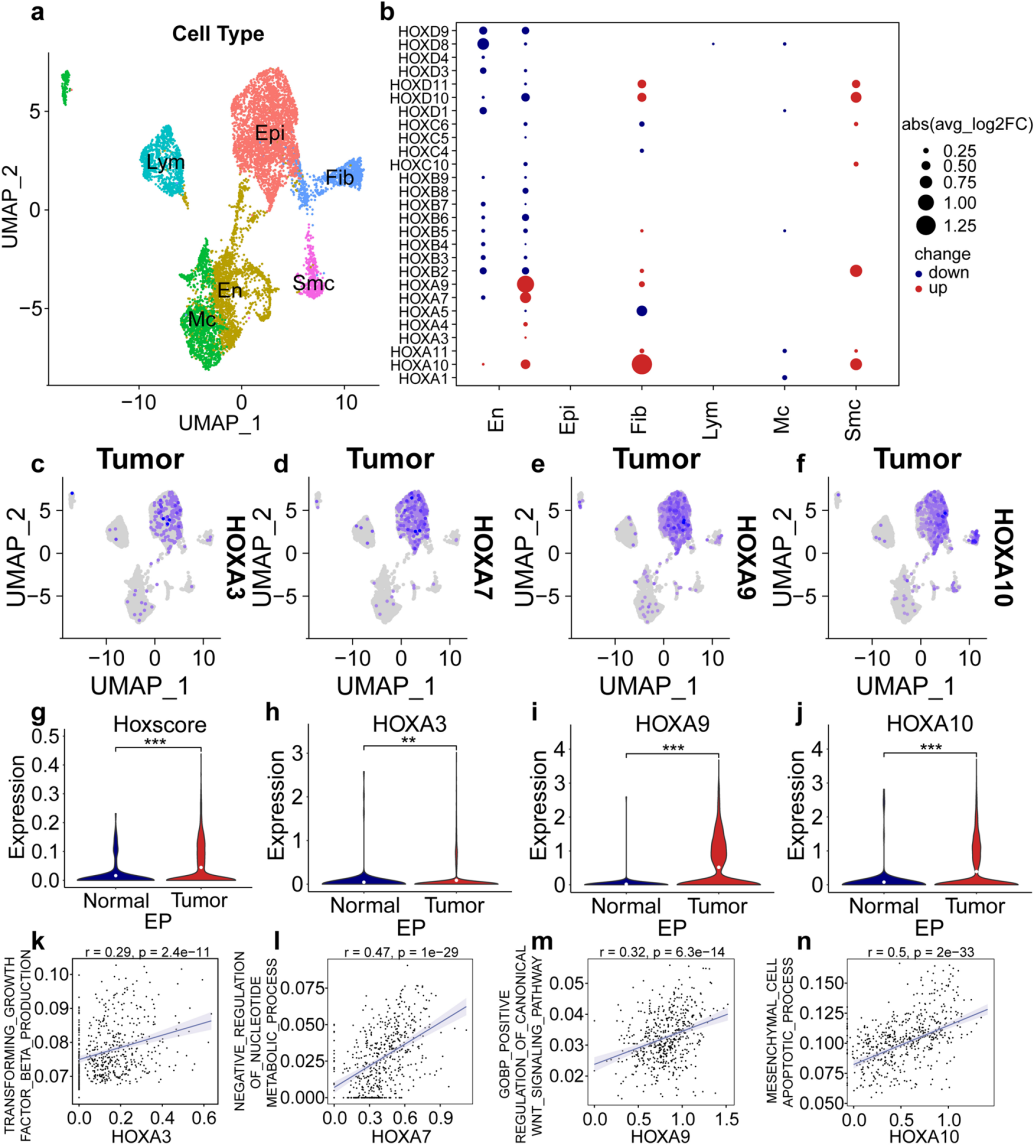
scRNA-seq of endometrial tissues. **a** UMAP graph showing the expression of the six cell types, including epithelial cells, endothelial cells, macrophages, lymphocytes, fibroblasts, and smooth muscle cells between normal and tumor samples. **b** Differences in the expression of *HOX* genes in the six cell types. The size of the dots represents the percentage of cells expressing the gene. The color of the dots represents the average expression level; red indicates high expression, whereas blue indicates low expression. **c**-**f** UMAP scatter diagram exhibiting the expression of *HOXA 3*, *HOXA 7, HOXA 9*, and *HOXA 10* in epithelial cells in tumor samples. **g** Differences of *HOX* score in epithelial cells between normal and tumor tissues (*P* < 0.05, Log-rank test) **h**-**j** Differences in the expression of *HOXA 3*, *HOXA9* and *HOXA 10* in epithelial cells between normal and tumor tissues (*P* < 0.05, Log-rank test). **k-n** Correlation between the *HOXA 3*, *HOXA 7*, *HOXA 9*, *HOXA 10*, and Gene Ontology Biological Process

To further explore cellular diversity and *HOX* gene expression in endometrial tissue, the six subjects enrolled were divided into a UCEC group (three patients with UCEC receiving surgery or other treatments) and a non-UCEC group (three subjects who underwent hysterectomy) (Table 1). Age distribution, gestation, and pregnancy were not different between the groups. We employed scRNA-seq technology. This analysis covered one normal and one tumorous tissue samples. Our results, obtained through clustering analysis, identified six main cell types within the tissue, with epithelial cells being the most dominant (Fig. 5a). We then investigated the differences in *HOX* gene expression among these cell types. Notably, several genes, including *HOXA* 3, *HOXA* 4, *HOXA* 7, *HOXA* 9, and *HOXA* 10, showed high expression levels in epithelial cells (Fig. S10b). The most significant expression differences were observed for *HOXA* 3, *HOXA* 4, *HOXA* 7, *HOXA* 9, and *HOXA* 10 (Fig. 5b). Subsequently, we analyzed the expression of these five *HOX* genes in epithelial cells between the normal and tumor tissue. The UMAP scatter diagram revealed that in epithelial cells, the expression of *HOXA* 3, *HOXA 4*, *HOXA* 7, *HOXA* 9, and *HOXA* 10 were expressed more in tumor tissues (Figs. 5c-f and S10c) than in normal tissues (Figs. S10d-h). Further analysis of the *HOX* score and gene expression differences for *HOX* genes in epithelial cells indicated that both were significantly higher in the tumor tissue compared to that in normal tissue (Figs. 5g-j and S10i-j). Finally, we explored the relationship between these four *HOX* genes and various biological pathways. We discovered a positive correlation between *HOXA* 3 and the Gene Ontology Biological Process (GOBP) for the transforming growth factor β product, between *HOXA 4* and the GOBP for the positive regulation of oxidative stress induced cell death, between *HOXA* 7 and the GOBP for the negative regulation of nucleotide metabolic process, between *HOXA* 9 and the GOBP for the positive regulation of canonical Wnt signaling pathway, and between *HOXA* 10 and the GOBP for the mesenchymal cell apoptotic process (Figs. 5k-n and S10k). These findings suggest that tumor occurrence and development may be linked to cellular signaling pathway dysfunctions caused by *HOX* gene expression.

**Table 1:** Comparison of basic data from eligible selected UCEC patients and controls. Including age, gestation, pregnancy, tumor size, histology, histological grading, Bokhman typing, FIGO staging, lymph node metastasis and treatment method

Patient		Ca1	Ca2	Ca3	N1	N2	N3
**Characteristic**	**Age**	58	56	67	46	54	61
	**Gestation**	4	3	1	0	5	3
	**Pregnancy**	3	1	1	0	3	2
	**Tumor size (cm)**	2.5*2.1*1.3	1.5*1.0*0.5	3.0*2.0*1.6	-	-	-
	**Histology**	Adenocarcinoma	Adenocarcinoma	Adenocarcinoma	-	-	-
	**Histological grading**	G1	G1	G2	-	-	-
	**Bokhman typing**	I	I	I	-	-	-
	**FIGO staging**	II	IA	IIIC1	-	-	-
	**Lymph node metastasis**	negative	negative	positive	-	-	-
	**Treatment method**	Surgery Radiotherapy	surgery	Surgery Chemotherapy Radiotherapy	-	-	-

a: Patients in the experimental group were annotated as Ga1, Ca2 and Ca3, respectively. Patients in the control group were labeled as N1, N2 and N3, respectively

b: Histological grading is divided into four levels, G1, G2, G3 and G4

c: Bokhman Classification divides UCEC into two types according to the clinical pathological characteristics and prognosis. Type I is oestrogen-dependent, and the type II is non-oestrogen-dependent

d: International Federation of Gynecology and Obstetrics (FIGO)

### Effect of *HOX* gene on CAFs in UCEC and construction of a prognostic model

**Fig. 6:**
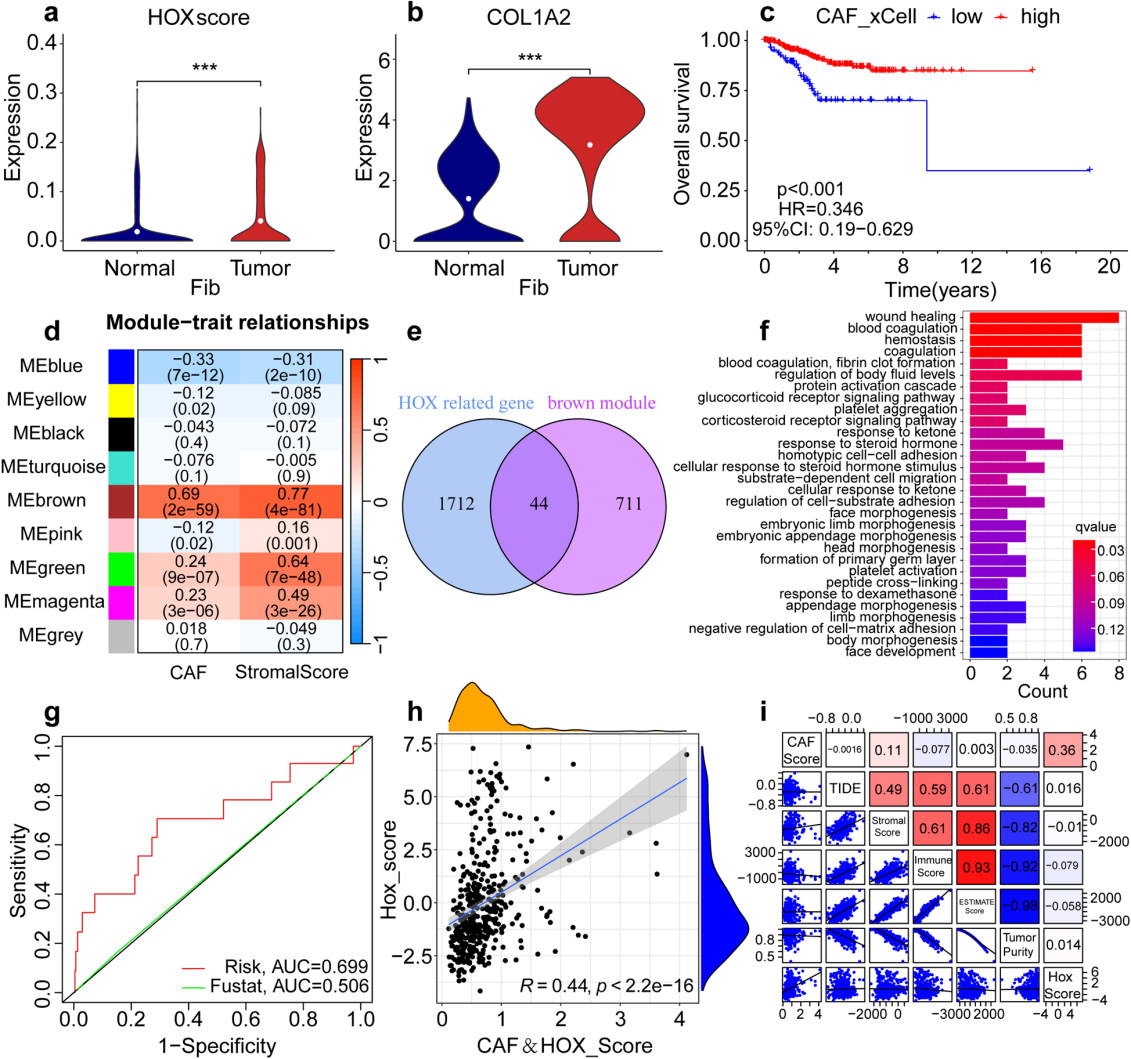
Combination of CAFs with *HOX* scores to establish a risk prognostic model. **a** Differences in the *HOX* score in fibroblasts between normal and tumor tissues (*P* < 0.05, Log-rank test). **b** Differences in the expression of COL1A2 gene in fibroblasts between normal and tumor tissues (*P* < 0.05, Log-rank test). **c** The survival curves of different CAF groups were estimated using the Kaplan–Meier plotter. (*P* < 0.001, Log-rank test). **d** The heatmap profiling the correlations between module genes, CAFs, and stromal score. **e** In the Venn diagram, 44 intersecting genes are shown between the *HOX*-related and brown module genes. **f** KEGG functional enrichment analysis of 44 intersecting genes. **g** ROC curve based on the prognostic risk model. **h** The correlation between the *HOX* score and CAF&*HOX* Score. **i** Analysis of the correlation of CAF scores

CAFs are one of the most abundant cell types in the TMEs. To further understand the role of CAFs in UCEC, the fibroblasts were examined in normal endometrial tissue and tumor tissue by scRNA-seq technology and found the higher *HOX* score in tumor tissue than in the normal group (Fig. 6a). And The *COL1A2* gene*,* as the marker gene for fibroblasts, was presented more higher expression in the tumor tissue (Fig. 6b). The Correlation analysis showed a positive association between *HOX* score and *COL1A2* gene (Fig. S10l). Next, we divided the samples derived from the TCGA database into high and low CAF score groups and found that patients in the high score group had significantly better survival than those in the low score group (Fig. 6c). Therefore, we used immune-related genes concentrated in UCEC to identify the key modules associated with CAFs using Weighted correlation network analysis (WGCNA). After constructing a similar clustering module through dynamic hybrid cutting, nine modules were identified. The Pearson’s correlation coefficients between ME and CAFs of each module were calculated. The brown module showed the highest positive correlation with CAFs (Fig. 6d). Subsequently, 44 genes intersecting *HOX*-related genes and Brown module-related genes were examined (Fig. 6e). To infer biologically interpretable results, using Metascape's functional enrichment analysis capability, the several most significantly enriched ontology terms were combined to annotate the putative biological roles of the 44 intersecting genes, such as angiogenesis, Protein Interaction Database integrin β1 pathway (PID integrin 1 pathway), cytokine signaling in lmmune system, regulation of synapse organization and calmodulin induced events (Fig. S11a). To explore the relationship between biological behaviors and intersecting gene expression in UCEC, we performed GO and KEGG enrichment analyses of the 44 intersecting genes. KEGG analysis revealed that these genes were enriched in multiple pathways, including substrate-dependent cell migration, regulation of cell substrate adhesion, and negative regulation of cell matrix adhesion (Fig. 6f). GO enrichment analysis showed that the intersecting genes were enriched in extracellular matrix structural constituents, basement membrane, wound healing, and blood coagulation (Fig. S11b). These pathways play a regulatory role in tumors, further illustrating the role of the 44 intersecting genes in the formation and development of tumors.

Next, based on results of the Cox proportional hazards model (Cox) analysis, 7 of the 44 intersecting genes were screened and found to be significantly associated with OS of patients with endometrial carcinoma (Fig. S11c). A prognostic risk scoring model was established using Least absolute shrinkage and selection operator (LASSO) regression analysis, and 10-fold cross-validation was used to train the model. According to lambda 1se, 0.01 was determined as an appropriate λ value (Fig. S11d). Finally, six non-zero coefficient UCEC genes (*IL17RE, MAPRE2, DCLK2, FIBIN, FBXO17*, and *NCAM1*) were obtained and used in survival analysis to verify their prognostic value and to further guide clinical treatment (Fig. S11e). To validate the predictive power of this prognostic risk model, we used receiver operating characteristic (ROC) curves. The results showed an Area Under Curve (AUC) of 0.699 for the risk score (Fig. 6g). The AUCs for 1-, 3-, and 5-year OS in the UCEC cohort were 0.699, 0.684, and 0.652, respectively (Fig. S11f). These data revealed the accuracy of the CAF/*HOX* score as a risk model for UCEC prognosis.

To verify the relationship between *HOX* scores and CAFs, we performed a correlation analysis. Scatter plots showed positive correlations between *HOX* scores and CAF expression (Fig. 6h). Finally, to assess the potential relationship between *HOX* scores and scores representing six functional states, including the CAF score, TIDE, stromal score, immune score, ESTIMATE score, and tumor purity, we conducted a series of Pearson correlation analyses. The results showed that the *HOX* score was positively associated with TIDE and tumor purity, but a negative correlation was observed between *HOX* scores and stromal, immune, and ESTIMATE scores (Fig. 6i). The results showed that *HOX* scores were related to the formation of CAFs in some ways and created a favorable environment for tumor development.

Overall, the prognostic risk model established by combining *HOX* score with CAFs appears to be a more complete and accurate scheme that can better guide the clinical treatment of individual patients in the future.

### *HOX* gene upregulated in endometrial tumor tissues compared to normal

**Fig. 7:**
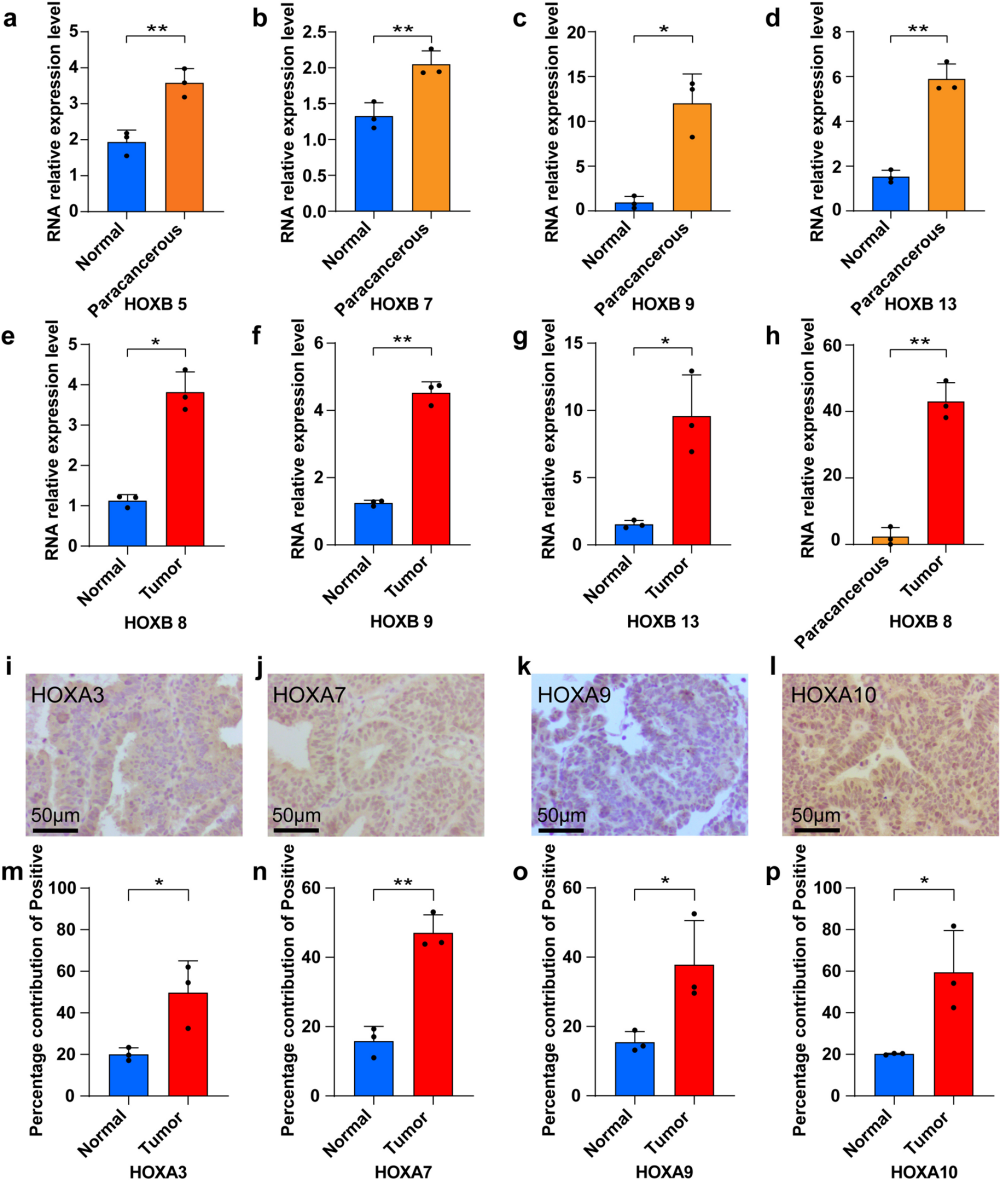
The expression of *HOX* genes was verified using RT-qPCR and IHC. **a**-**h** Levels of mRNA expression of *HOX* genes. The mRNA expression levels of *HOXB 5*, *HOXB 7*, *HOXB 8*, *HOXB 9*, and *HOXB 13* in endometrial, paracancerous, and normal endometrial tissues were measured using RT-qPCR. GAPDH was used as a loading control. Data are presented as the mean ± SD of triplicate independent experiments. *P* values were obtained using the Student’s t-test (**a-d**: control group: normal endometrial tissue; experimental group: paracancerous tissue. **e**–**g** control group: normal endometrial tissue; experimental group: endometrial tumor tissue. **h** control group: paracancerous tissue; experimental group: endometrial tumor tissue. *, *P* < 0.05; **, *P* < 0.01; ***, *P* < 0.001). **i**-**l** IHC for detecting the expression of *HOX* genes in tumor tissues (bar = 50 μm). Each experiment was performed in triplicate. **m**-**p** Percentage of positive staining for *HOX* gene (control group: normal endometrial tissue; experimental group: endometrial tumor tissue. *, *P* < 0.05; **, *P* < 0.01; ***, *P* < 0.001)

To further validate whether expression of the *HOX* gene set is generalized at the molecular level in the tissues of patients with UCEC, hematoxylin and eosin (H&E) staining, RT-qPCR and IHC were performed on normal endometrial tissues and tumor tissues. We performed H&E staining and RT-qPCR for three patients with UCEC (including endometrial tumor and paracancerous tissues) and three healthy individuals (normal endometrial tissues) (Table 1). H&E staining for the normal endometrial tissue confirmed endometrial histologic characteristics (Figs. S11g-i). H&E staining revealed an increased cell number, disordered arrangement, and increased mitotic figures in the tumor tissues, which was consistent with the diagnosis of UCEC (Figs. S12a-c). IHC revealed *PTEN* expression in normal endometrial cells (Fig. S12d). While the absence of *PTEN* expression in the tumor cells (Figs. S12e). These experiments confirmed histological differences between normal endometrial and endometrial tumor samples. Next, we performed RT-qPCR on the six samples. In the experimental group, expression of *HOXB 5, HOXB 7, HOXB 9,* and *HOXB 13* genes was generally higher than that in the normal endometrial tissue (*P* = 0.0024, *P* = 0.002, *P* = 0.0284, and *P* = 0.0029, respectively) (Figs. 7a-d). We also observed that the expression of *HOXB 8, HOXB 9*, and *HOXB 13* genes in UCEC tissues was generally higher than that in normal tissues (*P* = 0.0106, *P* = 0.0041, and *P* = 0.0373, respectively) (Figs. 7e-g). Additionally, *HOXB 8* expression was approximately 20 times higher in tumor tissues than in paracancerous tissues (*P* = 0.0017) (Fig. 7h). Next, IHC for fifteen normal endometrial tissues and fifteen tumor tissues was also performed using different *HOX* gene antibodies. The number of samples used to test for each gene was six samples (three normal endometrial tissues and three endometrial tumor tissues). All subjects underwent hysterectomy. Age distribution was not different between the groups (Table S1-5). We found that compare to the normal tissues, *HOXA 3*, *HOXA 4*, *HOXA 7*, *HOXA* 9, and *HOXA 10* showed stronger expression on the cytoplasm and nucleus in tumor tissues (Figs. 7i-l and S12f-k). The bar chart showed that in the tumor tissues, the percentage contribution of positive for *HOX* genes were higher than that in the normal group (Figs. 7m-p and S12l). These results showed that *HOX* expression levels gradually increased from normal tissues to tumor tissues, which further verified that *HOX* can act as a pro-oncogenic gene to regulate the occurrence and development of UCEC.

## Discussion

In this study, we analyzed the correlation of all *HOX* genes with UCEC. We then utilized unsupervised clustering and principal component analysis to construct *HOX* scores and combined them with clinical features and multiple biological pathways to identify a positive correlation between *HOX* score and UCEC. Using scRNA-seq, we identified six major cell types in our endometrial tumor samples, namely epithelial cells, endothelial cells, macrophages, lymphocytes, fibroblasts, and smooth muscle cells. We selected epithelial cells and fibroblasts with the higher *HOX* expression. Next, we examined the differential expression of *HOX* within epithelial cells between normal and tumor tissues, and explored the potential relationship between *HOX* genes and signaling pathways within epithelial cells. In addition, we compared the difference in the *HOX* score in CAFs between normal and tumor tissues. We explored the correlation of *HOX* scores and CAFs, and established a risk prognosis model based on the interaction between *HOX* genes and CAFs. We also found that the *HOX* gene can regulate some tumor-associated signaling pathways in CAFs. Finally, we collected thirty-six samples (eighteen normal and eighteen endometrial tumor tissues), and tested the expression of the *HOX* gene using RT-qPCR and IHC.

*HOX* genes, central regulators in living organisms, yielded transcription factors that exerted a paradoxical effect on tumorigenesis [4]. Their expression profiles had been implicated in both restraining the metastatic potential of cancer cells [9] and fueling tumor proliferation, migration, and invasion [10]. These genes were widely expressed across various solid tumor types, encompassing colorectal, lung, and squamous cell carcinomas [9, 16, 31]. Yet, the investigation into the interplay between *HOX* gene expression and immune cell infiltration within the TME remained under-explored. A precedent study highlighted a significant correlation between elevated *HOX*C6 expression and the expression of chemokines that recruit T cells, the degree of immune cell infiltration, and the presence of immune checkpoint markers in colorectal cancer [16]. Such findings underscored the indispensable role of *HOX* expression in inflammation, immunity, and tumor progression. Nevertheless, the literature on the association between *HOX* expression and UCEC was scant. The influence of the TME on tumor dynamics had been firmly established, with evidence suggesting that infiltrating immune cells can identify and eliminate neoplastic cells within the TME [11]. For instance, the activation of natural killer T (NKT) cells by α-galactosylceramide was shown to alter the composition of M1 macrophages and effector Th1 cells in secondary lymphoid organs and the TME, effectively curbing tumor growth [32]. However, the majority of contemporary studies concentrate on singular cell types within the TME, leaving the collective impact of multiple *HOX* genes on TME infiltration patterns largely unappreciated. Given the ambiguity surrounding the role of *HOX* expression in the etiology and evolution of UCEC, coupled with the dearth of understanding regarding *HOX*-mediated immune infiltration, a holistic evaluation of the prognostic utility and functional annotations of *HOX* genes in UCEC was warranted. Deciphering the function of *HOX* genes in immune cell infiltration provided a foundational framework for advancing our comprehension of the dialogue between *HOX* genes and antitumor immune responses, paving the way for tailored therapeutic interventions for individuals diagnosed with UCEC.

In this comprehensive analysis, we scrutinized the clinical data and transcriptomic profiles of 401 UCEC patients, focusing on the expression patterns of 39 *HOX* genes. Our findings delineated three distinct immune phenotypes within the cohort. Cluster A emerged as an immune-inflamed subtype, characterized by robust adaptive immune activation. Conversely, cluster B was typified by immune suppression, whereas cluster C presented as an immune-excluded phenotype, marked by heightened innate immune responses and stromal activation. With regard to the immune inflammatory phenotypes, often denoted as "hot" tumors, we observed that CD8 + T cells could infiltrate the tumor parenchyma, effectively engaging with neoplastic cells at the core. In contrast, the immune-excluded and immune-desert phenotypes, categorized as "non-inflamed" tumors, displayed a higher density of CD8 + T cells confined to the tumor periphery, rather than penetrating the tumor's substance. Particularly in immune-desert phenotypes, CD8 + T cell infiltration was virtually absent. Our data further revealed that Cluster C exhibited a pronounced stromal activation profile, encompassing upregulated pathways involved in angiogenesis, epithelial-mesenchymal transition (EMT), and transforming growth factor β (TGF-β) signaling. By integrating the TME cell-infiltrating features and survival outcomes across the clusters, we validated the accuracy of our immune phenotype classification, which was contingent upon distinct *HOX* expression signatures. The analysis of TME cellular infiltration patterns associated with varying *HOX* expression revealed that Cluster C was associated with a poorer prognosis, attributed to the suppression of immune cell activity. Moreover, a comparative assessment of *HOX* expression levels between endometrial carcinoma tissues and healthy counterparts showcased a degree of heterogeneity. This suggested that an imbalance in *HOX* gene expression may serve as a critical determinant in the etiology and progression of UCEC, and the intricate relationship between *HOX* gene expression, immune phenotyping and the tumor microenvironment in UCEC underscored the potential of *HOX* genes as biomarkers and therapeutic targets for personalized cancer care.

In our investigation, we uncovered that the disparities in mRNA transcriptomes, delineated by distinct *HOX* expression patterns, were profoundly intertwined with *HOX* genes themselves and immune-related biological processes. These DEGs, deemed as *HOX*-related signature genes, played a pivotal role in defining two genomic subtypes that bore significant relevance to matrix activation and immune reactions. These findings resonated with the clustering outcomes based on *HOX* expression, reinforcing the notion that *HOX* expression profoundly impacts the configuration of the TME. From these observations, we deduced that an exhaustive appraisal of *HOX* expression patterns could significantly enrich our insights into the TME's cellular infiltration traits. However, recognizing the limitations of our current analytical approach in precisely forecasting individual patient expression patterns, and taking into account the intrinsic heterogeneity of *HOX* gene expression, the imperative need for quantifying *HOX* patterns within individual tumors became evident. To address this exigency, we devised a scoring system dedicated to evaluating the *HOX* expression profiles in patients diagnosed with endometrial carcinoma. This system revealed that the immune-excluded phenotype was associated with a higher *HOX* score, while the immune-inflamed phenotype was characterized by a lower *HOX* score. This disparity suggested that the *HOX* score serves as a dependable and potent instrument for comprehensively gauging *HOX* expression patterns within individual tumors. Furthermore, it could facilitate the determination of TME infiltration patterns, also recognized as tumor immunophenotypes. Comparative analyses of mRNA transcriptomes amongst patients with variant *HOX* scores unveiled substantial correlations with biological pathways intimately linked to *HOX* genes and immunity. Importantly, the *HOX* score demonstrated superior evaluative capabilities concerning patient clinical attributes, encompassing tumor differentiation grade, mutational load, age, survival status, and overall clinical prognosis. The correlation between the *HOX* score, tumor stage, and prognosis was strikingly significant. Integrated analyses corroborated the potential of the *HOX* score to operate as an autonomous prognostic biomarker for endometrial carcinoma. This finding underscored its utility in clinical decision-making, offering a promising avenue for personalized medicine in UCEC management.

TMB and MSI are increasingly recognized as pivotal biomarkers for predicting the efficacy of immunotherapy in cancer patients [33, 34]. Our analysis disclosed a striking positive correlation between the *HOX* score and TMB, suggesting a potential synergistic effect on patient outcomes. When we cross-referenced the *HOX* score with TMB, we observed that patients bearing high *HOX* and TMB scores enjoyed the most favorable survival outcomes, whereas those with high *HOX* scores paired with low TMB experienced the least survival benefit. Contrary to initial expectations, patients exhibiting low *HOX* scores in conjunction with high TMB levels did not demonstrate the most significant survival advantage. This unexpected result could potentially be attributed to the limited sample size in our study. Nevertheless, our data consistently revealed that patients in the low *HOX* score cohort fared better prognostically, irrespective of their tumor burden status. The integration of *HOX* scores with TMB in survival analysis offered more detailed and precise prognostic insights. Moreover, we integrated the *HOX* score with TIDE and physical fitness levels, demonstrating that the *HOX* score could serve as a novel predictive marker for the effectiveness of ICIs. Our findings underscored the significant role of *HOX* expression in the development of distinct matrix environments and immune landscapes within the TME, alongside its impact on immune cell infiltration. Immunomodulatory molecules like Programmed Death Receptor-1 (PD-1) are key regulators of immune responses, typically expressed on activated T cells, NK cells, B-cells, and subsets of myeloid cells [35]. Dysregulation of checkpoint gene expression and function is a cardinal mechanism implicated in various diseases [36, 37]. Our results indicated that the *HOX* score was intricately linked to disrupted immune checkpoints, potentially enhancing responsiveness to immunotherapeutic interventions. The amalgamation of a *HOX* gene signature with a repertoire of integrated biomarkers, encompassing mutation load, neoantigen load, PD-L1 expression, stromal and immune TME characteristics, and MSI status, emerges as a potent predictive tool for immunotherapy efficacy. This integrative approach holds considerable promise for guiding personalized therapeutic strategies in future clinical practice, optimizing patient selection and treatment outcomes in the realm of immunotherapy.

The aberrant expression of *HOX* genes can lead to dysregulation in certain tumor-related signaling pathways, which is one of the decisive factors contributing to the development of UCEC. Among different *HOX* patterns, repair pathways such as nucleotide excision repair, homologous recombination, and mismatch repair are significantly upregulated in cluster C compared to cluster A and B. These DNA damage repair pathways can be persistently activated when nucleotide sequences mutate, enhancing repair capacity and regulating the cell cycle to maintain genomic stability and integrity [38, 39]. The p53 signaling pathway, as a protective response, can be triggered by DNA damage, oxidative stress, and other stimuli. It activates genes involved in DNA repair, such as *XRCC1* and *APEX1*, which encode proteins that help repair DNA damage and prevent further genomic mutations [40, 41]. Through scRNA-seq, we find that *HOX* genes are highly expressed in UCEC epithelial cells and fibroblasts, and their expression regulates typical pathways associated with tumor invasion and metastasis in these two cell types. In epithelial cells, the expression of *HOX* genes is significantly positively correlated with processes such as TGF-β, mesenchymal cell apoptosis, and Wnt signaling pathways. Studies have shown that due to metabolic imbalance in tumor cells, reactive oxygen species can activate TGF-β, inducing EMT and increasing malignancy [42]. Mesenchymal cells can induce tumor cell death and inhibit the migratory activity of tumor cells [43]. However, under the regulation of *HOX*-mediated mesenchymal cell apoptosis, the number of tumor cells can increase, weakening their inhibitory effect on invasion. Abnormal activation of the Wnt signaling pathway may lead to an expansion of the stem cell pool and the generation of cancer stem cells, promoting tumor formation and recurrence. In fibroblasts, *HOX*-induced modulation of cell–matrix adhesion pathways can alter the mobility and invasiveness of tumor cells. For example, reduced expression of E-cadherin in tumor cells decreases intercellular adhesion, promoting EMT and conferring stronger migratory capabilities [44]. The interaction between tumor cells and vascular endothelial cell adhesion molecules induces angiogenesis, supplying necessary nutrients and stimulating proliferation and invasion of tumor cells [45]. During the activation of wound healing pathways, certain protein factors such as vascular endothelial growth factor (VEGF) and TGF-β can be secreted [46, 47]. These factors may increase the malignancy of tumors by inducing EMT and other mechanisms [42]. Collectively, these results posited that *HOX* genes may serve as target genes within specific signaling pathways resident in epithelial cells and fibroblasts, culminating in the development of UCEC. This insight underscores the potential of targeting *HOX* genes as a therapeutic strategy to disrupt the signaling pathways that underpin UCEC progression.

CAFs represent a crucial element within the TME, playing a multifaceted role in cancer progression. These cells secrete a plethora of growth factors, inflammatory mediators, and extracellular matrix components [48], which collectively foster tumor cell proliferation, confer resistance to therapy, and orchestrate immune evasion [49]. In our investigative approach, leveraging scRNA-seq technology, we elucidated the prominent expression of *HOX* genes within CAFs. This finding was further substantiated by a positive correlation between the *HOX* scores and the presence of CAFs. To decipher the genetic architecture underlying this association, we employed WGCNA to pinpoint modules of co-expressed genes that were intimately tied to CAF activity. By integrating *HOX*-related genes with these module genes, we narrowed down to a set of intersecting, prognostic-relevant genes. The development of a risk model based on these genes demonstrated a commendable predictive capability for estimating the 1-, 3-, and 5-year overall survival rates of patients. This validation not only underscored the precision and clinical significance of the *HOX* score as an independent biomarker for survival prediction but also hinted at its potential as a prognostic tool in diverse cancer types.

To complement the theoretical and computational analyses conducted in our study, we reinforced our findings with empirical validation through RT-qPCR and IHC. The RT-qPCR results unequivocally demonstrated that the expression levels of *HOX* genes were significantly elevated in endometrial tumor tissues compared to the corresponding normal tissue controls. This quantitative molecular evidence provided a robust confirmation of our bioinformatic predictions, substantiating the differential expression of *HOX* genes in cancerous versus healthy endometrial tissue. Furthermore, the application of IHC corroborated our molecular findings again. IHC staining revealed a markedly increased positive rate of *HOX* expression within tumor tissues, visually depicting the enhanced presence of *HOX* proteins in neoplastic cells. This qualitative data, in conjunction with the quantitative RT-qPCR results, served to strengthen the validity of our theoretical studies. Together, these experimental approaches—RT-qPCR for gene expression quantification and IHC for protein localization—yielded convergent evidence supporting the central role of *HOX* genes in UCEC. The integration of these methodologies allowed us to establish a comprehensive understanding of *HOX* gene expression patterns, confirming their upregulation in tumor samples and reinforcing the clinical relevance of our computational discoveries. These findings underscored the importance of *HOX* genes in the pathogenesis of UCEC, creating the way for potential therapeutic strategies targeting these critical regulatory molecules. The combination of theoretical studies with experimental validation provided a solid foundation for future translational research aimed at developing targeted therapies for patients with UCEC.

Our research substantiated that the landscape of *HOX* gene expression orchestrates extensive mechanisms within the UCEC TME, and a comprehensive appraisal of *HOX* expression patterns within individual tumors enhanced our understanding of cellular infiltration characteristics within the TME. Concurrently, through the development of a *HOX* scoring system to elucidate the role of *HOX* genes in cancer, we offered a holistic evaluation of *HOX* profiles and TME cellular infiltration features in individual patients, facilitating the identification of tumor immune phenotypes and guiding clinical practice more effectively. Furthermore, within the epithelial cells and CAFs of UCEC, *HOX* genes served as target genes for a multitude of tumor-related signaling pathways. This positioning potentially made them critical molecular markers for the advancement of future precision medicine approaches.

In summary, the *HOX* score demonstrated independent prognostic power for UCEC, potentially serving as a predictive biomarker for patient survival. Our comprehensive analysis of *HOX* provided a novel and insightful perspective to the field of UCEC research, contributing to a better understanding of its epigenetics and immunotherapeutic landscape.

## Methods

### Dataset source and preprocessing

Public gene expression data and complete clinical annotation were retrieved from TCGA and ICGC. We selected patients with available survival information for further evaluation and excluded the others. In total, 401 eligible tumor samples from the TCGA-UCEC cohort and 128 eligible tumor samples from the ICGC-UCEC cohort were included for subsequent analysis. All samples were used in the mutation analysis. The samples from the TCGA-UCEC cohort were used in unsupervised clustering and consensus clustering analyses. RNA sequencing data, expressed as Fragments Per Kilobase of exon model per Million mapped fragments (FPKM) values of gene expression were downloaded from the Genomic Data Commons (GDC, https://portal.gdc.cancer.gov/) using the R packages TCGA and ICGC biolinks. These packages were specifically developed for integrative analysis with GDC data [50]. Additionally, somatic mutation data were acquired from the TCGA database. The dataset from TCGA-UCEC was downloaded for CNV analysis. Data were analyzed using R (version 4.2.3) and R Bio conductor packages.

### Unsupervised clustering and consensus clustering analysis

We extracted a total of 39 *HOX* related genes from the TCGA and ICGC datasets to identify distinct modification patterns mediated by these genes. The analyzed genes included *HOXA 1, HOXA 2, HOXA 3, HOXA 4, HOXA 5, HOXA 6, HOXA 7, HOXA 9, HOXA 10, HOXA 11, HOXA 13, HOXB 1, HOXB 2, HOXB 3, HOXB 4, HOXB 5, HOXB 6, HOXB 7, HOXB 8, HOXB 9, HOXB 13, HOXC 4, HOXC 5, HOXC 6, HOXC 8, HOXC 9, HOXC 10, HOXC 11, HOXC 12, HOXC 13, HOXD 1, HOXD 3, HOXD 4, HOXD 8, HOXD 9, HOXD 10, HOXD 11, HOXD 12*, and *HOXD 13.* Unsupervised clustering analysis was applied to identify distinct patterns of *HOX*-related genes based on their expression. This analysis helped classify patients for further analysis. The number of clusters and their stability were determined using a consensus clustering algorithm [51]. The ConsensuClusterPlus package was used to perform these analyses, with 1,000 repetitions conducted to ensure the stability of the classifications [52].

### Enrichment gene set variation analysis with functional annotation

We conducted GSVA enrichment analysis using the “GSVA” R package to explore differences in biological processes among *HOX* patterns. GSVA is a non-parametric and unsupervised method widely used to estimate variation in pathway activity and biological processes across samples in an expression dataset [53]. For this analysis, gene sets titled “c2.cp.kegg.v6.2.-symbols” were sourced from the MSigDB database. Statistical significance was determined with an adjusted *P*-value of less than 0.05. The clusterProfiler R package was used to perform functional annotation for *HOX*-related genes, with the cutoff value of false discovery rate (FDR) set at less than 0.05.

### TME cell infiltration estimation

To quantify the relative abundance of each cell type infiltrating the UCEC TME, we employed the single-sample gene-set enrichment analysis (ssGSEA) algorithm. The gene set used to mark each type of TME infiltrating immune cell was obtained from the study by Charoentong, which includes various human immune cell subtypes including activated CD8^+^ T cells, activated dendritic cells, macrophages, natural killer T cells, and regulatory T cells, among others [54, 55]. The ssGSEA-derived enrichment scores were used to represent the relative abundance of each TME infiltrating cell in each sample. Additionally, we calculated tumor purity, stromal, immune, and ESTIMATE scores for each sample using the ESTIMATE algorithm. This algorithm assesses the tumor component in each sample [56]. Subgroup comparisons were analyzed using the “limma” R package, allowing for a detailed examination of score variations.

### Identification of DEGs between distinct *HOX* gene phenotypes

To explore the diversity among *HOX* genes-related phenotypes, we classified patients into three distinct groups based on *HOX* gene expression. We used the empirical Bayesian approach provided by the “limma” R package to identify DEGs between different patterns [57]. The criteria for determining significant DEGs were set with an adjusted *P*-value threshold of less than 0.001. Further analysis on the pathways associated with these DEGs was conducted using GO and KEGG databases.

### Design and generation of *HOX* gene score

To evaluate individual patient *HOX* patterns in UCEC, we developed a scoring system called the *HOX* score. The process to establish the *HOX* gene signature involved several key steps:

Initially, DEGs identified from various *HOX* clusters were normalized across all UCEC samples. Overlapping genes were then extracted. We used an unsupervised clustering method to analyze these overlapping DEGs, classifying the patients into several groups for more detailed analysis. A consensus clustering algorithm was employed to determine the number of gene clusters and assess their stability.

Next, each gene within the signature underwent a prognostic evaluation using a univariate Cox regression model. Genes showing significant prognostic impact were selected for further analysis. We then applied PCA to construct the *HOX*-relevant gene signature. The first two principal components (PC1 and PC2) were used as the signature scores as follows:

$$HOX\;\mathrm{score}\:=\:\mathrm\Sigma(\mathrm{PC}1_{\mathrm i}\:+\:\mathrm{PC}2_{\mathrm i})$$

where i is the expression of *HOX* cluster-related genes.

### Correlation between the *HOX* gene score and other related biological processes or clinical features

We performed a correlation analysis to further reveal the association between the *HOX* gene signature and some related clinical features, including (1) immune-checkpoints such as CD 44, CD 96, TIGIIT and others; (2) microsatellite status, such as MSS, MSI-L, and MSI-L; (3) TMB; (4) TIDE; (5) age; (6) tumor grade; (7) several drugs including cyclopamine, fedratinib, pazopanib, foretinib, and others; (8) ESTIMATES score; (9) immune score; (10) stromal score; (11) tumor purity; and (12) IPS.

### Weighted gene co-expression network analysis and prognostic model construction

WGCNA is a method established for studying biological networks and disease correlations. We used the R package “WGCNA 16” to perform WGCNA, aiming to identify key modules related to CAFs using immuno-related genes within the TCGA-UCEC dataset. To identify the genes linked to prognosis, we employed forest plots for dichotomous variables and LASSO regression. The predictive value of the constructed prognostic model was evaluated using ROC curves.

### Tissue selection

Endometrial tissues were collected from eighteen patients with endometrial tumors and eighteen with normal endometrial conditions at the first hospital of Shanxi Medical University. All patients underwent hysterectomy. The selection of the tissues was based on histopathological analyses performed by a pathologist with over two years of clinical experience. Each tissue sample measured 1.0 cm in length, 0.5 cm in width, and 0.5 cm in thickness. Paracancerous tissues were excised 1 cm from the tumor margin. Six tissues were subjected to hematoxylin and eosin staining and RT-qPCR. IHC was performed on paraffin-embedded thirty tissue samples. One tumor tissue and one normal tissue were selected from the collected samples for scRNA-seq.

### scRNA-seq

Raw reads from human endometrial cell fastq files were processed using the Cell Ranger Software Suite (10 × Genomics Cell Ranger 4.0.0), with refdata-gex-GRCh38-2020-A as the reference for mapping reads to the human genome (GRCh38/hg38). This processing generated unique molecular identifier (UMI) matrices [58]. The outputs from Cell Ranger were then imported into Seurat using the ‘Read10X’ function [59]. Quality control measures included removing cells with UMI counts in the upper 10% for each sample to eliminate potential outliers. Additionally, cells with fewer than 500 UMIs detected or those with more than 40% mitochondrial UMIs were filtered out. Genes expressed in fewer than 1 cell were also removed. Quality control was managed using the ‘Seurat’ package (version 4.0). For normalization, a global scaling method called LogNormalize was applied to normalize gene expression measurements across cells, considering both characteristic and total expression levels. To reduce the computational load and minimize noise, PCA was employed for initial dimensionality reduction. Visualization of the annotated cellular information within the dataset was achieved using uniform manifold approximation and projection.

### Immunohistochemistry

Proportion of antibody dilution: *HOXA 3* (1: 200) (ab230879, Abcam lnc, Cambridge, MA, USA), H*OXA 4* (1: 200) (ab131049, Abcam lnc, Cambridge, MA, USA), *HOXA 7* (1: 200) (ab211521, Abcam lnc, Cambridge, MA, USA), *HOXA 9* (1: 500) (ab140631, Abcam lnc, Cambridge, MA, USA), *HOXA 10* (1: 200) (ab191470, Abcam lnc, Cambridge, MA, USA). 4-μm-thick tissue sections were dewaxed and dehydrated by xylene and a graded ethanol series, respectively. Then, endogenous peroxidase activity was blocked by 3% H_2_O_2_. Sections were incubated with primary antibodies for 4 h at room temperature, followed by incubation with the corresponding second antibody. Subsequently, tissue sections were stained with 3,3’-diaminobenzidine and hematoxylin. The positive rate of IHC was analyzed by Image J.

### RT-qPCR

Total RNA was extracted from tissue samples using Trizol reagent (Ambion, USA). The mRNA was then reverse transcribed into cDNA using the cDNA Synthesis SuperMix (TransGen Biotech, Beijing). PCR amplification was conducted using a PCR machine (Bioer Technology, GeneMax Tc-s-B, China) using the following cycling parameters: an initial denaturation at 95 ℃ for 5 min, followed by 40 cycles of denaturation at 95 ℃ for 10 s, and annealing at 60 ℃ for 30 s. Gene expression levels were quantified using the ΔΔCt method. The experiments were performed in triplicates to ensure reproducibility. The primers used for RT-qPCR were as follows: GAPDH forward 5'-GCTCTCTGCTCCTCCTGTTC-3', reverse 5'-ACGACCAAATCCGTTGACTC-3'; *HOXB* 5 forward 5'-CTCTGAGCGGCTCTTACAGG-3', reverse 5'-GCCCGGTCATATCATGGCT-3'; *HOXB 6* forward 5'-AGGACTGCAGCCCGATACTA-3', reverse 5'-CGAGATTGGGTTTTAGCTTTGC-3'; *HOXB 7* forward 5'-TGCGAAGCTCAGGAACTGAC-3', reverse 5'-CCAAAATTTCTCCTTTCTCCCTCC-3'; *HOXB 8* forward 5'-GCTGCCATGCAAGCTTAGAC-3', reverse 5'-GTCCGGCGGCTGCTTG-3'; *HOXB 9* forward 5'-GAGAGGCCGGATCAAACCAA-3', reverse 5'-GGGAGGACTGGGGGTAATCT-3'; *HOXB 13* forward 5'-TAAAACGCTTTGGATTCCCCC-3', reverse 5'-CCGCTGGAGTCTGCAAAT-3'.

### Statistical analysis

The correlation between TME infiltrating immune cells and the expression of *HOX* regulators was assessed using Spearman and distance correlation analyses. Cut-off points were established based on the correlation of the *HOX* score with patient survival within each dataset subgroup, using the surf R package. Survival curves for the prognostic analysis were generated using the Kaplan–Meier method. The significance of the differences was determined using a log-rank test. All statistical tests were two-sided, with significance set at *P* < 0.05. All data processing was performed using the R4.2.3 software.

### Supplementary Information

Supplementary Material 1: Fig. S1. (a) Survival difference between high and low gene expression in the pan-cancer, including DFI, DSS, OS, PFS. (b) Correlation of the *HOX* genes with tumor stage. (c) The role of *HOX* gene expression on pathways. Activate: red. Inhibit: blue. Fig. S2. Flowchart of data collection and analysis. Fig. S3. (a) 124 of the 529 (23.44%) patients with UCEC had genetic alterations in *HOX* genes. Each column represents an individual patient. The upper barplot shows the tumor mutation load, while the number on the right indicates the mutation frequency in each gene. The right barplot shows the proportion of each variant type. The stacked barplot below shows the fraction of conversions in each sample. (b) The CNV mutation frequency of *HOX* genes was prevalent. The column represents the alteration frequency: green dot indicates the deletion frequency; red dot indicates the amplification frequency. (c) The location of CNV alterations in *HOX* genes on chromosomes. (d) KEGG functional enrichment analysis of genes contained in the CNV. (e) GO functional enrichment analysis of *HOX* genes contained in the CNV. (f) Spearman correlation analysis of the studied *HOX* gene. Fig. S4. (a) Diagram of consensus clustering analysis. (b) Unsupervised clustering of *HOX* genes in the TCGA endometrial cancer cohort. The *HOX* cluster, tumor grade, survival status, sex, and age were used as patient annotations. Red indicates high, whereas blue represents low expression. (c) GSVA enrichment analysis showing the state of metabolic pathways in distinct patterns between A and B clusters. The heatmap was used to visualize these biological processes. Red indicates activated pathways, whereas blue represents inhibited pathways. (d) Diagram of consensus clustering analysis for the DEGs. (e) Principal component analysis for the two gene clusters. Fig. S5. Expression patterns of *HOX* gene in endometrial tumor patients and healthy individuals. Heatmap of differential expression genes between tumor and control groups; the *HOX* genes in each module were annotated; the line graph showed the trend in the gene module expression, the text on the right showed the enriched pathways for each module gene. Fig. S6. (a) The proportion of patients with different tumor grades in low or high *HOX* score groups. (b) The proportion of patients with different survival state in low or high *HOX* score groups. (c) The proportion of patients with different age in low or high *HOX* score groups. (d) Differences in *HOX* score among distinct age groups. (e–g) Alluvial diagram showing the changes of *HO*X clusters, gene cluster, *HOX* score and grade, survival status, age. Fig. S7. (a) The abundance of each TME infiltrating cell type among the three *HOX* gene patterns. The upper and lower ends of the boxes represent the interquartile range of values. The lines in the boxes represent the median value, whereas dots show the outliers. Asterisks represent the statistical *P* value (*P < 0.05; **P < 0.01; ***P < 0.001). (b) Heatmap showing the difference in immune cell infiltration between low and high *HOX* score groups. Red represents high expression, whereas blue represents low expression. Values represent the correlation strength. (c) Correlations between immune score and TME infiltrating cell types using Spearman analysis. Blue indicates negative correlation, whereas red represents positive correlation. Values represent the correlation strength. (d-f) Comparison of the relative distribution of immune cells including IFNG gene (d), myeloid-derived suppressor cells, (e) and CD8 + T-cells (f) between high and low *HOX* score groups. (g) The proportion of patients with different MSI in low and high *HOX* score groups. (h) Differences in the *HOX* score among three distinct MSI groups (*P* < 0.05, Log-rank test). Fig. S8. (a-d) The relative distribution of IPS between *HOX* score high and low groups (*P* = 0.13,Log-rank test). (e-l) The correlation between the *HOX* score and several immune check points, including CD244, CD96, TIGIT, CSF1R, BTLA, CTLA4, HAVCR2 and PDCD1. Fig. S9. (a) Unsupervised clustering of different immune cells and pathways. The *HOX* score, tumor purity, ESTIMATES score, Immune score, and Stromal score were used as patient annotations. Red represents high, whereas blue represents low expression. Values represent the correlation strength. (b-e) Violin plot exhibited the difference in ESTIMATESscore, Immune score, Stromal score and tumor purity between the low and high *HOX* score groups. (f-g) Differences in the IC_50_ differences of anti-tumor drugs between different *HOX* score groups. f: fedratinib. g: pazopanib. (h-i) Correlation between anti-tumor drugs and *HOX* scores. h: fedratinib. i: pazopanib. (j-l) the difference of IC_50_ of several anti-tumor drugs between the low and high *HOX* score groups (*P* < 0.001, Log-rank test). j.rapamycin. k:doxorubicin. l:thapsigargin. Fig. S10. (a) the difference of IC_50_ of nilotinib between the low and high *HOX* score groups (*P* < 0.001, Log-rank test). (b) The expression of *HOX* genes in the six cell types. Red indicates high expression, whereas blue represents low expression. (c) The UMAP scatter diagram exhibited the expression of *HOXA 4* in epithelial cells in tumor samples. (d-h) The UMAP scatter diagram exhibiting the expression of *HOXA 3*, *HOXA 4*, *HOXA 7, HOXA 9*, and *HOXA 10* in epithelial cells in normal samples. (i) Differences in the expression of *HOXA 4* in epithelial cells between normal and tumor tissues (*P* < 0.05, Log-rank test). (j) Differences in the expression of *HOXA 7* in epithelial cells between normal and tumor tissues (*P* < 0.05, Log-rank test). (k) Correlation between the *HOXA 4* and Gene Ontology Biological Process. (l) The correlation between the *HOX* score and *COL1A2* gene. Fig. S11. (a) Metascape visualization of the interactome network formed by the 44 intersecting genes. (b) GO functional enrichment analysis of 44 intersecting genes, including cellular component (CC), molecular function (MF) and biological process (BP). High expression, red. Low expression, blue. The length of the bar chart indicates the count of genes enriched. (c) Univariate Cox regression analysis was used to assess the genes that related to prognosis. (d) Feature selection was conducted using the LASSO regression model through tenfold crossvalidation and lambda 1se. Coefficient distribution plots were generated for the log (lambda) sequence. (e) LASSO non-zero coefficient 6 significant genes in UCEC. (f) ROC curve analyses in predicting 1-, 3-, and 5-year overall survival (OS) in the TCGA-UCEC cohorts. (g-i) H&E staining was performed to observe pathological changes in normal endometrial tissues (bar = 50 μm). Fig. S12. (a-c) H&E staining was performed to observe pathological changes tumor tissues (bar = 50 μm). (d-e) Differential immunohistochemical expression of PTEN between normal endometrial tissues and tumor tissues (d: normal; e: tumor) (bar = 50 μm). (f) Immunohistochemistry of the tumor tissue was performed against *HOX* gene. (g-k) Immunohistochemistry of the normal endometrial tissue was performed against *HOX* gene. (l) Percentage of positive staining for *HOX* gene (control group: normal endometrial tissue; experimental group: endometrial tumor tissue. *, *P* < 0.05; **, *P* < 0.01; ***, *P* < 0.001). Table S1 Comparison of basic data from eligible selected endometrial cancer patients and controls. Including age, tumor size, histology, histological grading. Table S2. Comparison of basic data from eligible selected endometrial cancer patients and controls. Including age, tumor size, histology, histological grading. Table S3. Comparison of basic data from eligible selected endometrial cancer patients and controls. Including age, tumor size, histology, histological grading. Table S4. Comparison of basic data from eligible selected endometrial cancer patients and controls. Including age, tumor size, histology, histological grading. Table S5. Comparison of basic data from eligible selected endometrial cancer patients and controls. Including age, tumor size, histology, histological grading.

## Data Availability

All data used in our study can be acquired from the cancer fenome Atlas (TCGA; https://www.genome.gov/) and the ICGC data portal (https://dcc.icgc.org/). Single-cell RNA sequencing gene expression data generated in this study has been deposited in the Annotare < EMBL-EBI database (https://www.ebi.ac.uk/fg/annotare/edit/18643/#DESIGN:PROTOCOLS). Any other data are available from the corresponding author on reasonable request. Software and resources used for analysis and plotting are described in each method section.

## Abbreviations

AUC: Area Under Curve
CAFs: Cancer-associated fibroblasts
CNV: Copy number variation
Cox: Cox proportional hazards model
CTL: Cytotoxic T lymphocytes
CTLA-4: Cytotoxic T lymphocyte-associated antigen-4
DEGs: Differentially expressed genes
DFI: Disease-free interval
DSS: Disease-specific survival
ESTIMATE: Estimation of STromal and Immune cells in MAlignant Tumour tissues using Expression data
FDR: False discovery rate
FPKM: Fragments Per Kilobase of exon model per Million mapped fragments
GDC: The Genomic Data Commons
GO: Gene Ontology
GSVA: Gene set variation analysis
H&E: Hematoxylin and eosin
HLA: Human leukocyte antigen
IC_50_: Half maximal inhibitory concentration
ICGC: International Cancer Genome Consortium database
ICI: Immune checkpoint inhibitors
iDCs: Immature dendritic cells
IFN: Interferon
IHC: Immunohistochemistry
IL-1: Interleukin-10
KEGG: Kyoto Encyclopedia of Genes and Genomes
LASSO: Least absolute shrinkage and selection operator
MDSCs: Myeloid-derived suppressor cells
miRNAs: MicroRNAs
MSI: Tumor microsatellite instability
MSS: Microsatellite stability
NK: Natural killer
OS: Overall survival
PCA: Principal component analysis
PD-1: Programmed death 1
PFS: Progression-free survival
ROC: Receiver operating characteristic
PID: Protein Interaction Database
RT-qPCR: Real-time quantitative PCR
scRNA-seq: Single-cell RNA sequencing
ssGSEA: Single-sample gene-set enrichment analysis
TCGA: The Cancer Genome Atlas database
TIDE: Tumor Immune Dysfunction and Exclusion
TLs: T lymphocytes
TMB: Tumor mutation burden
TME: Tumor microenvironment
UCEC: Endometrial cancer
VEGF: Vascular endothelial growth factor
WGCNA: Weighted correlation network analysis
