# Supplementary material for: Effects of HOX family regulator-mediated modification patterns and immunity characteristics on tumor-associated cell type in endometrial cancer
Source: Mol Biomed. 2024 Aug 14;5:32. doi: 10.1186/s43556-024-00196-w (PMC11322468; doi:10.1186/s43556-024-00196-w)
Supplement: Supplementary file 1 — Supplementary Material 1: Fig. S1. (a) Survival difference between high and low gene expression in the pan-cancer, including DFI, DSS, OS, PFS. (b) Correlation of the HOX genes with tumor stage. (c) The role of HOX gene expression on pathways. Activate: red. Inhibit: blue. Fig. S2. Flowchart of data collection and analysis. Fig. S3. (a) 124 of the 529 (23.44%) patients with UCEC had genetic alterations in HOX genes. Each column represents an individual patient. The upper barplot shows the tumor mutation load, while the number on the right indicates the mutation frequency in each gene. The right barplot shows the proportion of each variant type. The stacked barplot below shows the fraction of conversions in each sample. (b) The CNV mutation frequency of HOX genes was prevalent. The column represents the alteration frequency: green dot indicates the deletion frequency; red dot indicates the amplification frequency. (c) The location of CNV alterations in HOX genes on chromosomes. (d) KEGG functional enrichment analysis of genes contained in the CNV. (e) GO functional enrichment analysis of HOX genes contained in the CNV. (f) Spearman correlation analysis of the studied HOX gene. Fig. S4. (a) Diagram of consensus clustering analysis. (b) Unsupervised clustering of HOX genes in the TCGA endometrial cancer cohort. The HOX cluster, tumor grade, survival status, sex, and age were used as patient annotations. Red indicates high, whereas blue represents low expression. (c) GSVA enrichment analysis showing the state of metabolic pathways in distinct patterns between A and B clusters. The heatmap was used to visualize these biological processes. Red indicates activated pathways, whereas blue represents inhibited pathways. (d) Diagram of consensus clustering analysis for the DEGs. (e) Principal component analysis for the two gene clusters. Fig. S5. Expression patterns of HOX gene in endometrial tumor patients and healthy individuals. Heatmap of differential expression genes betw [file 43556_2024_196_MOESM1_ESM.pdf]

ORIGINAL RESEARCH

JinPeng Li et al

Effects of *HOX* family regulator-mediated modification patterns and immunity characteristics on tumor-associated fibroblasts and epithelial cells in endometrial cancer

JiaoLin Yang[1,#], JinPeng Li[2,#], SuFen Li[1,#], YuTong Yang[2], HuanCheng Su[1], HongRui Guo[1], Jing Lei[1], YaLin Wang[1], KaiTing Wen[1], Xia Li[1], SanYuan Zhang[1,*], Zhe Wang[1,*]

[1]Department of Gynecology, First Hospital of Shanxi Medical University, Taiyuan 030001, China.

[2]Shanxi Medical University, Taiyuan 030001, China.

[#]These authors have contributed equally to this work and share first authorship.

* Correspondence:

SanYuan Zhang

zsyprofessor@sxmu.edu.cn

Zhe Wang

wangzhe@sxmu.edu.cn

Keywords: *HOX* gene, Endometrial cancer, Tumor microenvironment, scRNA-seq, CAFs.

Fig. S1

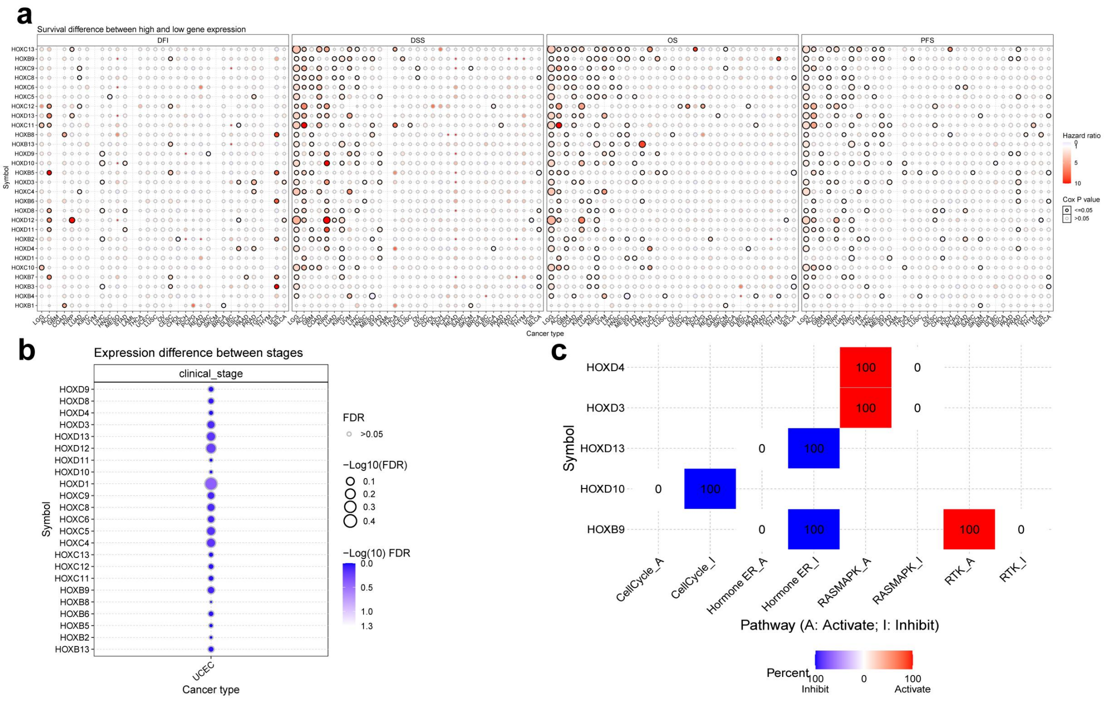

Fig. S1 (a) Survival difference between high and low gene expression in the pan-cancer, including DFI, DSS, OS, PFS. (b) Correlation of the *HOX* genes with tumor stage. (c) The role of *HOX* gene expression on pathways. Activate: red. Inhibit: blue.

Fig. S2

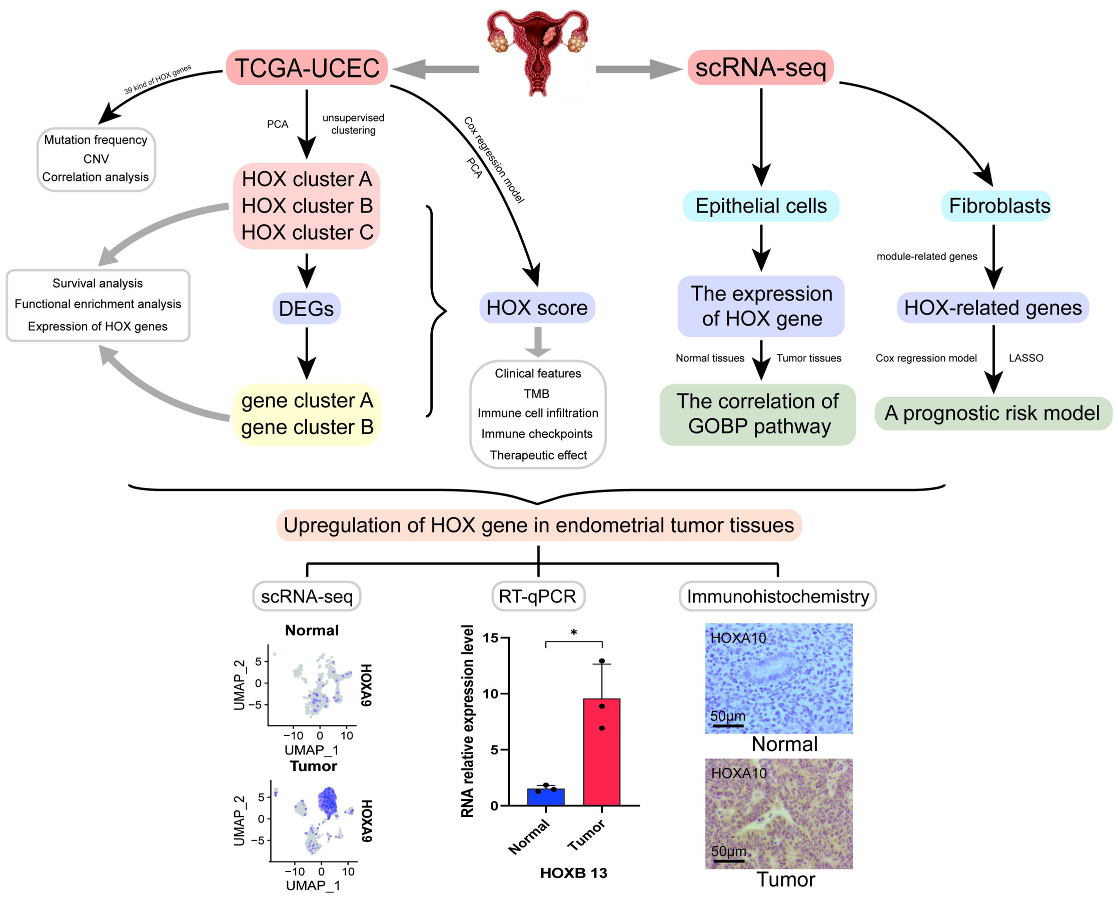

Fig. S2 Flowchart of data collection and analysis.

Fig. S3

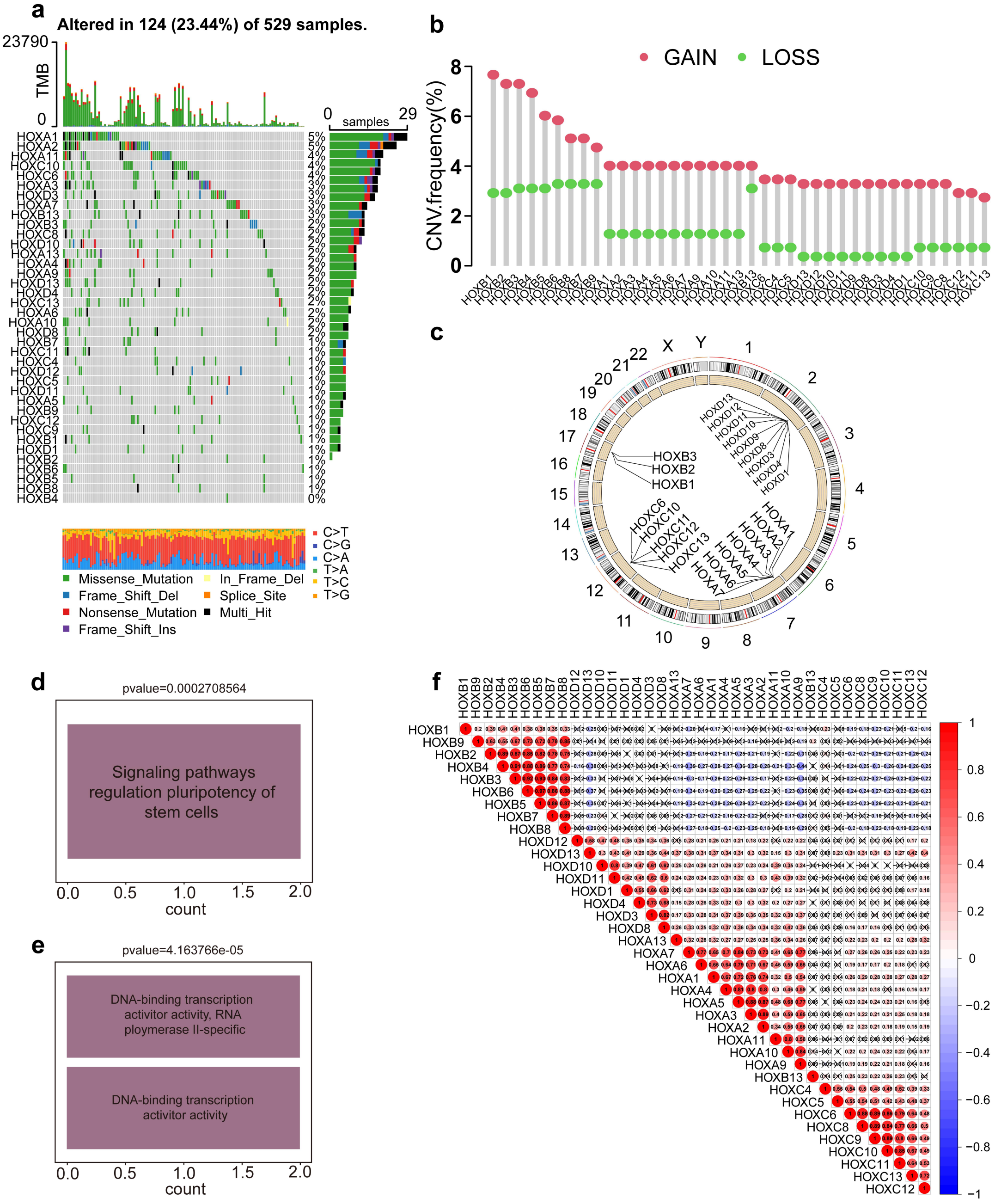

Fig. S3 (a) 124 of the 529 (23.44%) patients with UCEC had genetic alterations in *HOX* genes. Each column represents an individual patient. The upper barplot shows the tumor mutation load, while the number on the right indicates the mutation

frequency in each gene. The right barplot shows the proportion of each variant type. The stacked barplot below shows the fraction of conversions in each sample. (b) The CNV mutation frequency of *HOX* genes was prevalent. The column represents the alteration frequency: green dot indicates the deletion frequency; red dot indicates the amplification frequency. (c) The location of CNV alterations in *HOX* genes on chromosomes. (d) KEGG functional enrichment analysis of genes contained in the CNV. (e) GO functional enrichment analysis of *HOX* genes contained in the CNV. (f) Spearman correlation analysis of the studied *HOX* gene.

Fig. S4

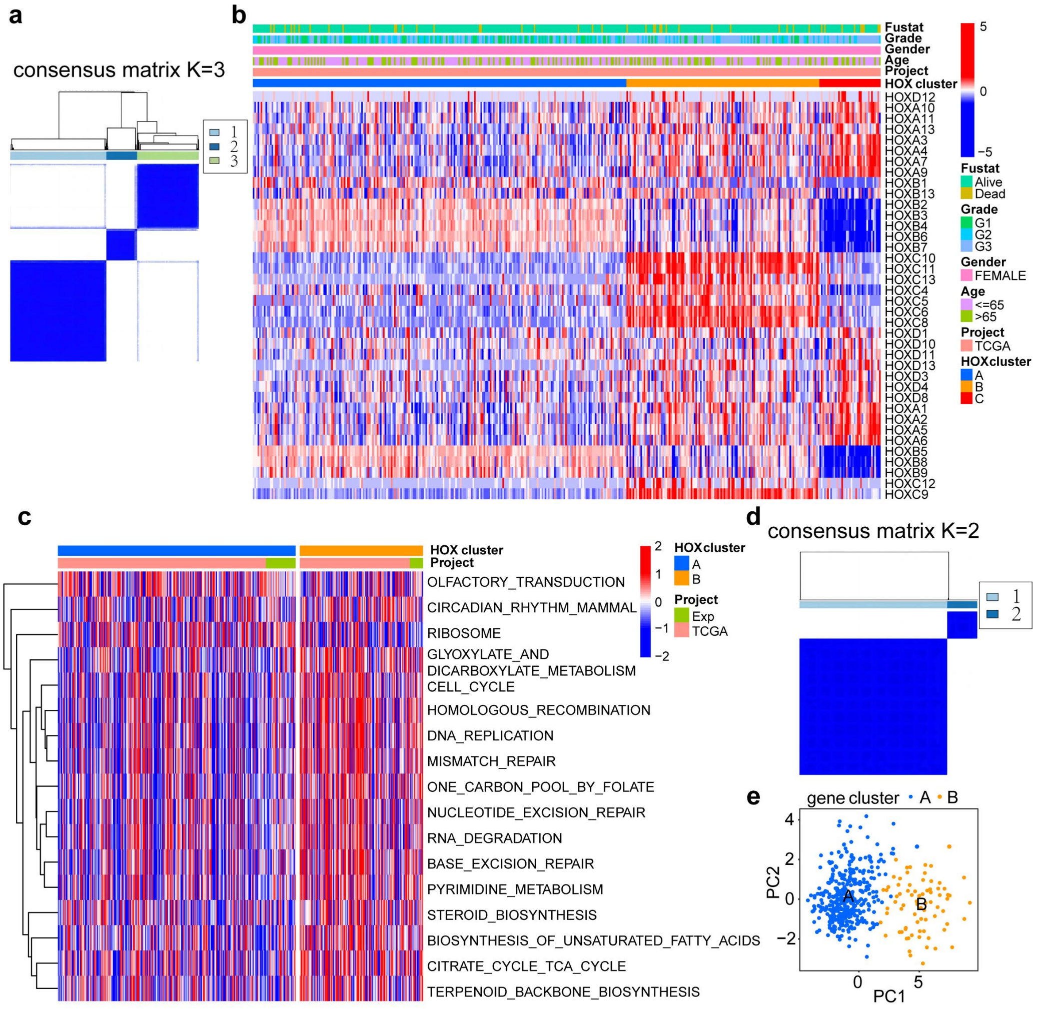

Fig. S4 (a) Diagram of consensus clustering analysis. (b) Unsupervised clustering of *HOX* genes in the TCGA endometrial cancer cohort. The *HOX* cluster, tumor grade, survival status, sex, and age were used as patient annotations. Red indicates high, whereas blue represents low expression. (c) GSVA enrichment analysis showing the state of metabolic pathways in distinct patterns between A and B clusters. The heatmap was used to visualize these biological processes. Red indicates activated pathways, whereas blue represents inhibited pathways. (d) Diagram of consensus clustering analysis for the DEGs. (e) Principal component analysis for the two gene clusters.

Fig. S5

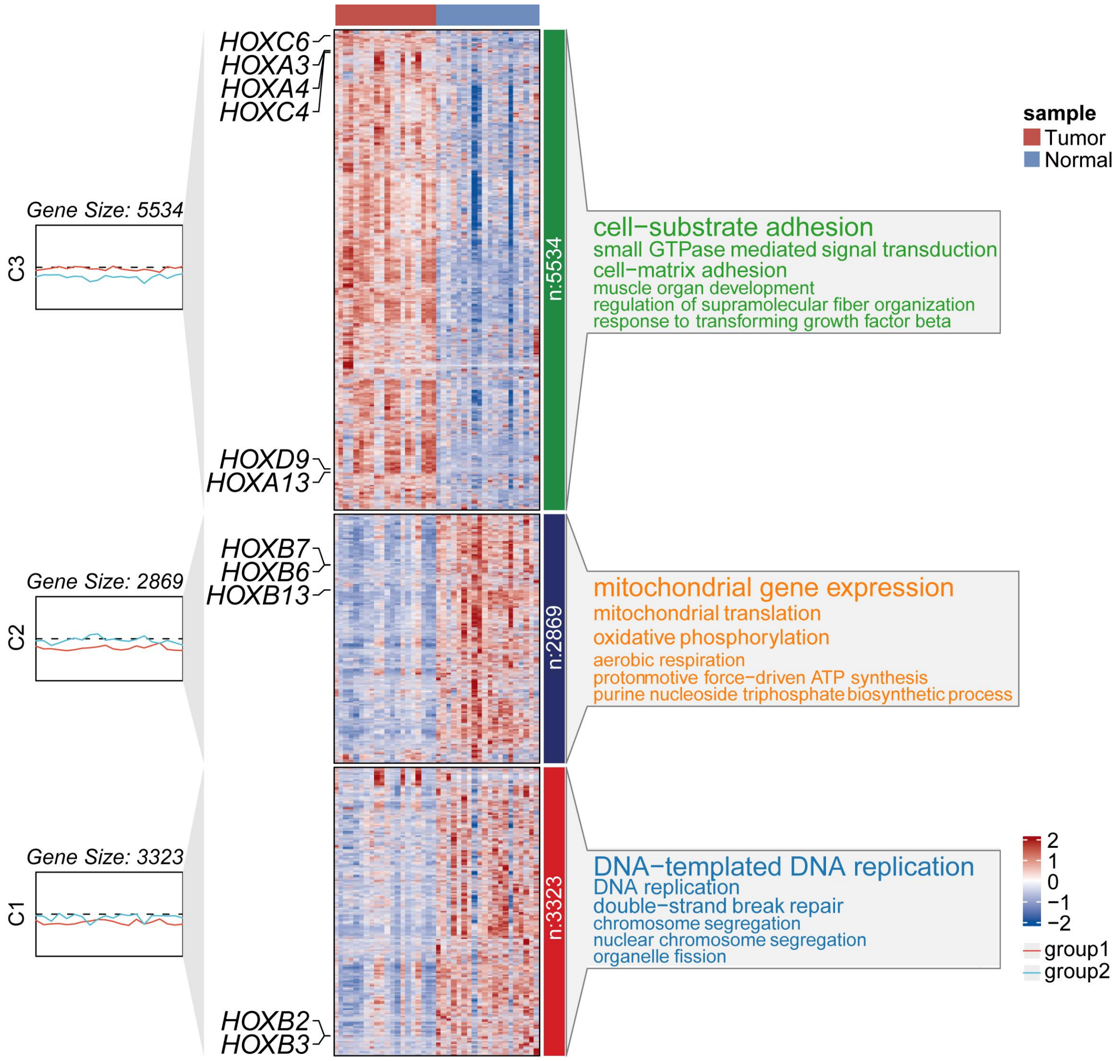

Fig. S5 Expression patterns of *HOX* gene in endometrial tumor patients and healthy individuals. Heatmap of differential expression genes between tumor and control groups; the *HOX* genes in each module were annotated; the line graph showed the trend in the gene module expression, the text on the right showed the enriched pathways for each module gene.

Fig. S6

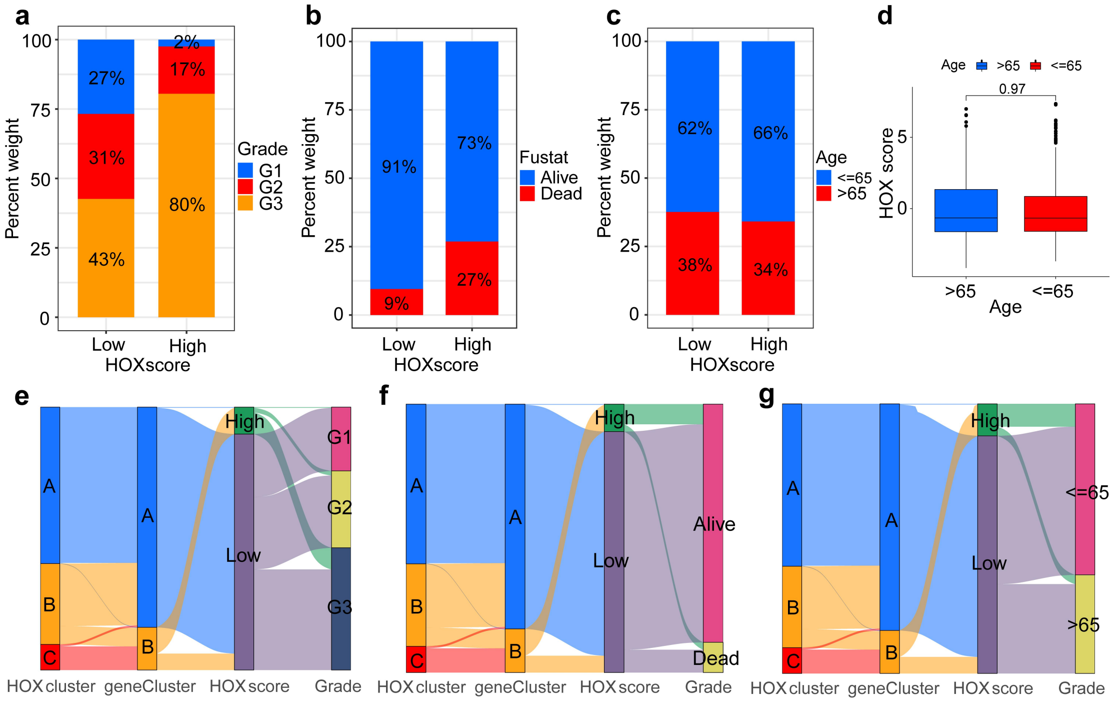

Fig. S6 (a) The proportion of patients with different tumor grades in low or high *HOX* score groups. (b) The proportion of patients with different survival state in low or high *HOX* score groups. (c) The proportion of patients with different age in low or high *HOX* score groups. (d) Differences in *HOX* score among distinct age groups. (e-g) Alluvial diagram showing the changes of *HOX* clusters, gene cluster, *HOX* score and grade, survival status, age.

Fig. S7

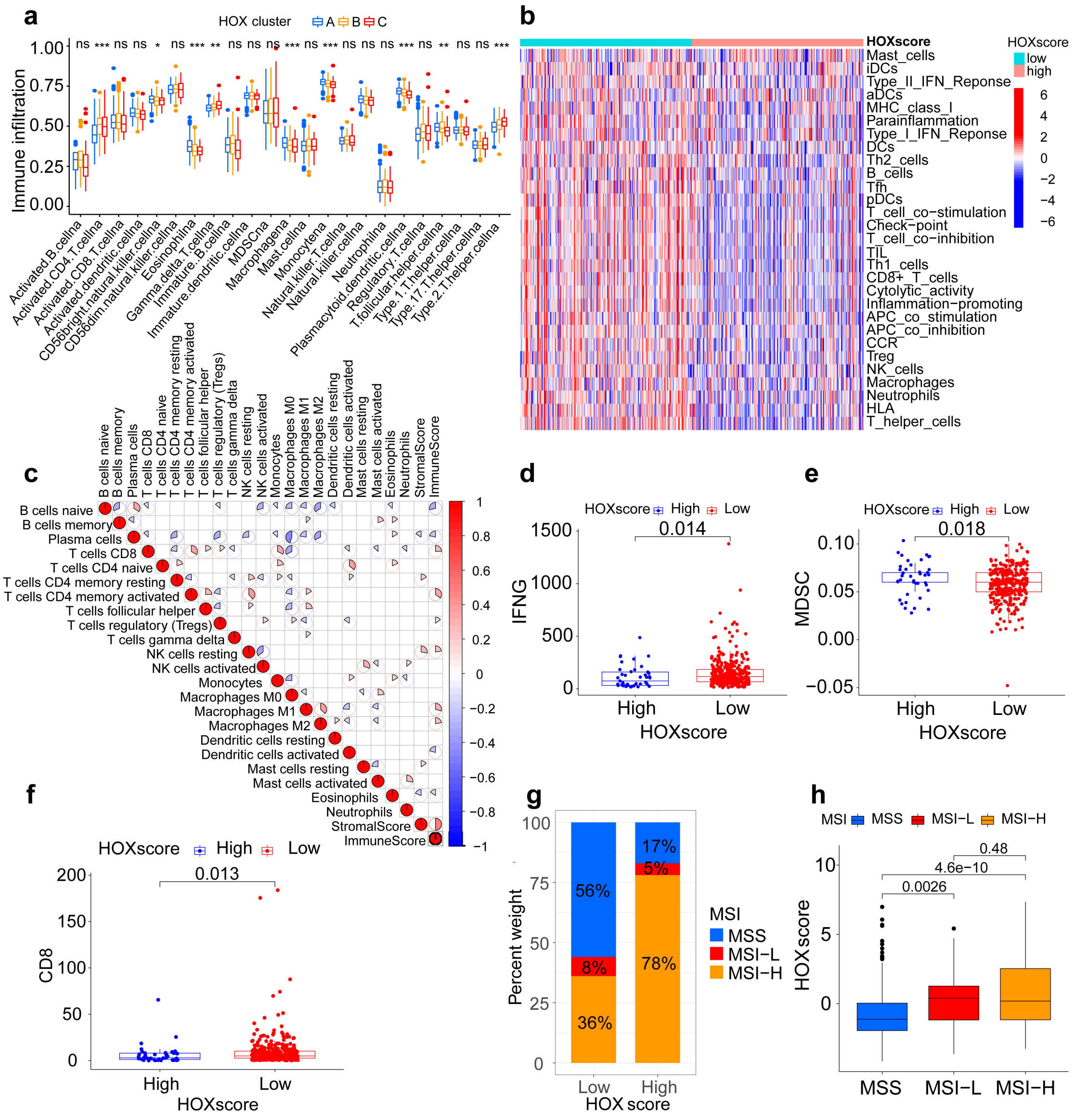

Fig. S7 (a) The abundance of each TME infiltrating cell type among the three *HOX* gene patterns. The upper and lower ends of the boxes represent the interquartile range of values. The lines in the boxes represent the median value, whereas dots show the outliers. Asterisks represent the statistical P value (*P < 0.05; **P < 0.01; ***P < 0.001). (b) Heatmap showing the difference in immune cell infiltration between low and high *HOX* score groups. Red represents high expression, whereas blue represents

low expression. Values represent the correlation strength. (c) Correlations between immune score and TME infiltrating cell types using Spearman analysis. Blue indicates negative correlation, whereas red represents positive correlation. Values represent the correlation strength. (d-f) Comparison of the relative distribution of immune cells including IFNG gene (d), myeloid-derived suppressor cells, (e) and CD8+ T-cells (f) between high and low *HOX* score groups. (g) The proportion of patients with different MSI in low and high *HOX* score groups. (h) Differences in the *HOX* score among three distinct MSI groups ($P < 0.05$, Log-rank test).

Fig. S8

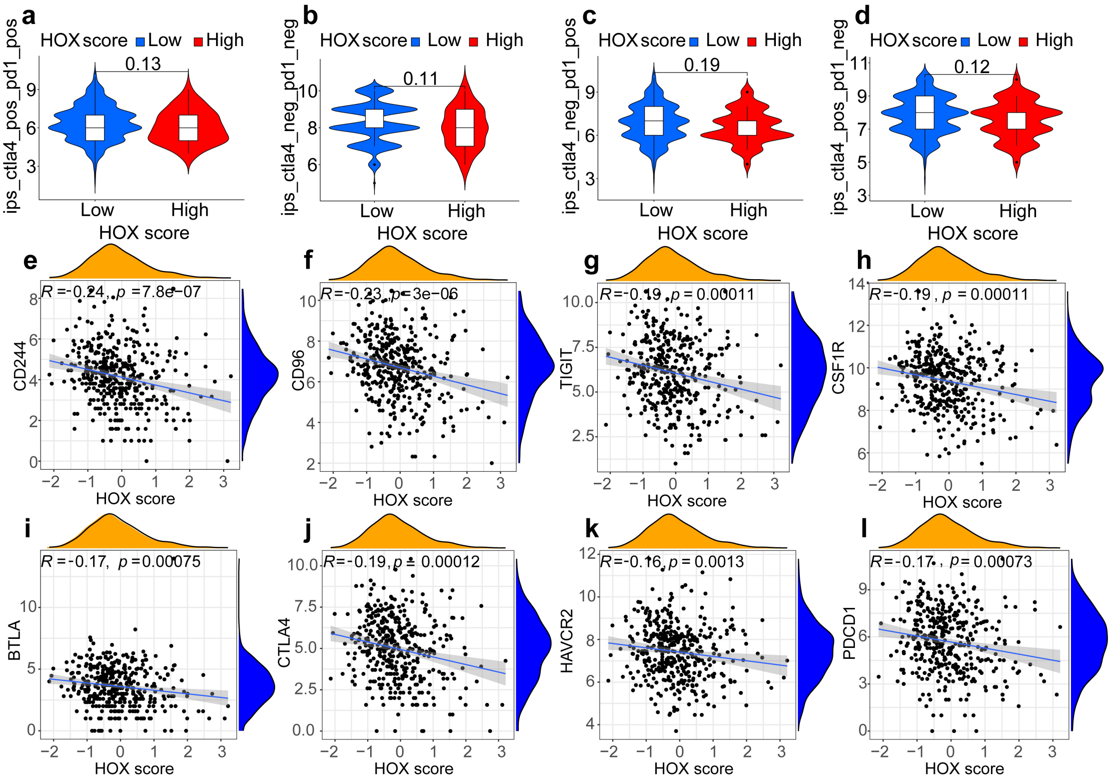

Fig. S8 (a-d) The relative distribution of IPS between *HOX* score high and low groups (*P*=0.13,Log-rank test). (e-l) The correlation between the *HOX* score and several immune check points, including CD244, CD96, TIGIT, CSF1R, BTLA, CTLA4, HAVCR2 and PDCD1.

Fig. S9

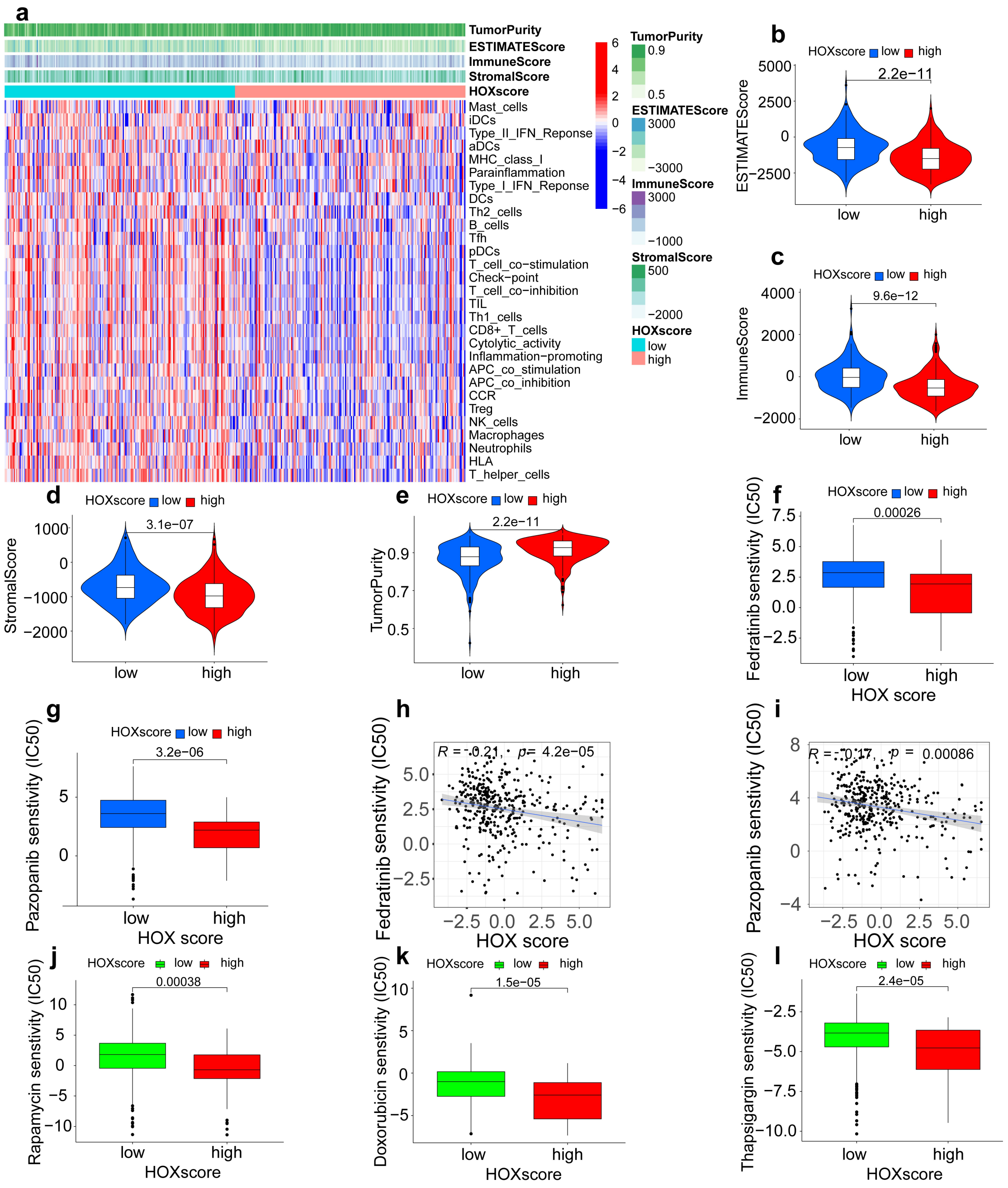

Fig. S9 (a) Unsupervised clustering of different immune cells and pathways. The *HOX* score, tumor purity, ESTIMATES score, Immune score, and Stromal score were used as patient annotations. Red represents high, whereas blue represents low expression. Values represent the correlation strength. (b-e) Violin plot exhibited the

difference in ESTIMATESscore, Immune score, Stromal score and tumor purity between the low and high *HOX* score groups. (f-g) Differences in the IC_{50} differences of anti-tumor drugs between different *HOX* score groups. f: fedratinib. g: pazopanib. (h-i) Correlation between anti-tumor drugs and *HOX* scores. h: fedratinib. i: pazopanib. (j-l) the difference of IC_{50} of several anti-tumor drugs between the low and high *HOX* score groups (P <0.001, Log-rank test). j.rapamycin. k:doxorubicin. l:thapsigargin.

Fig. S10

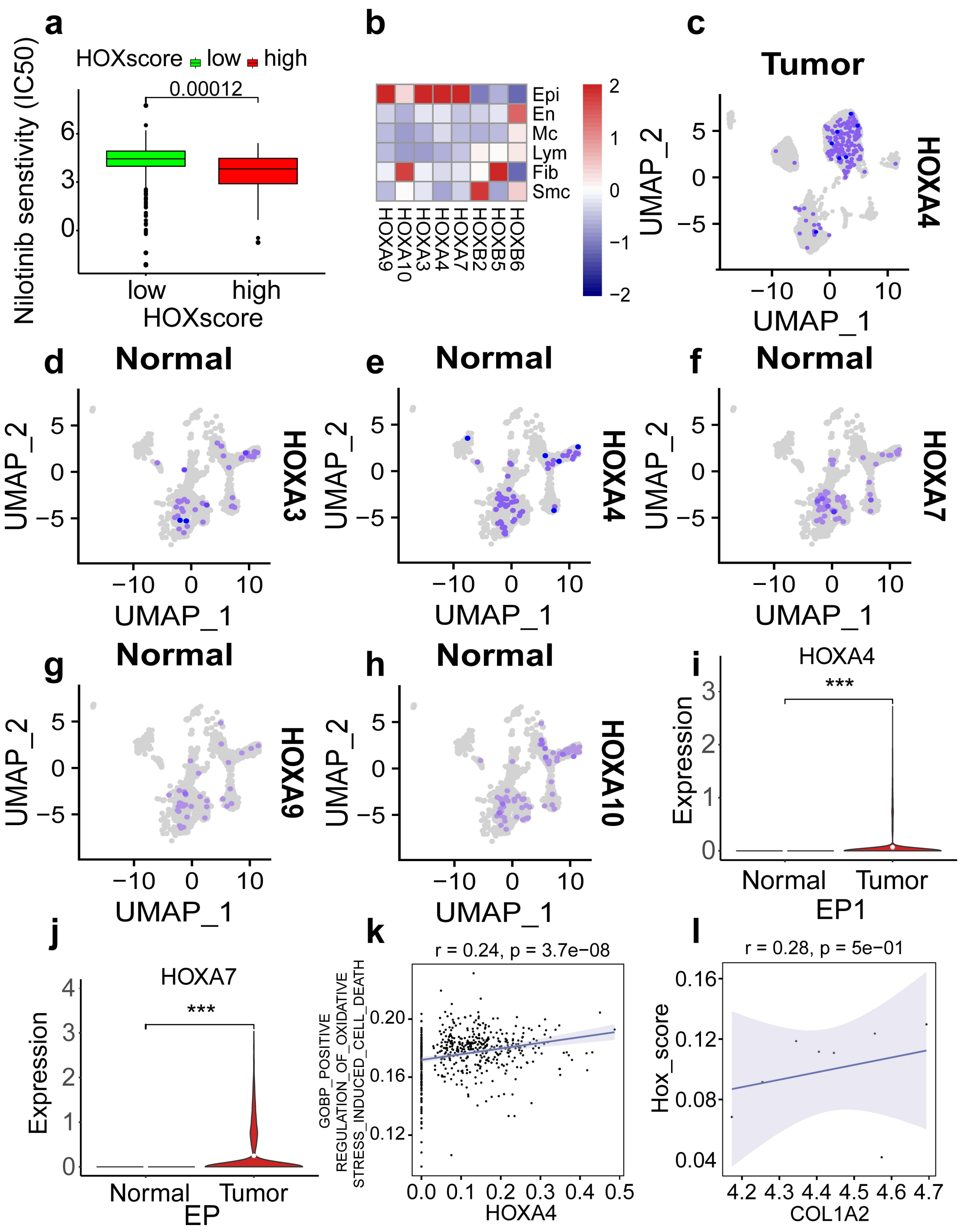

Fig. S10 (a) the difference of IC_{50} of nilotinib between the low and high *HOX* score groups (*P* <0.001, Log-rank test). (b) The expression of *HOX* genes in the six cell types. Red indicates high expression, whereas blue represents low expression. (c) The

UMAP scatter diagram exhibited the expression of *HOXA 4* in epithelial cells in tumor samples. (d-h) The UMAP scatter diagram exhibiting the expression of *HOXA 3*, *HOXA 4*, *HOXA 7, HOXA 9*, and *HOXA 10* in epithelial cells in normal samples. (i) Differences in the expression of *HOXA 4* in epithelial cells between normal and tumor tissues ($P < 0.05$, Log-rank test). (j) Differences in the expression of *HOXA 7* in epithelial cells between normal and tumor tissues ($P < 0.05$, Log-rank test). (k) Correlation between the *HOXA 4* and Gene Ontology Biological Process. (l) The correlation between the *HOX* score and *COL1A2* gene.

Fig. S11

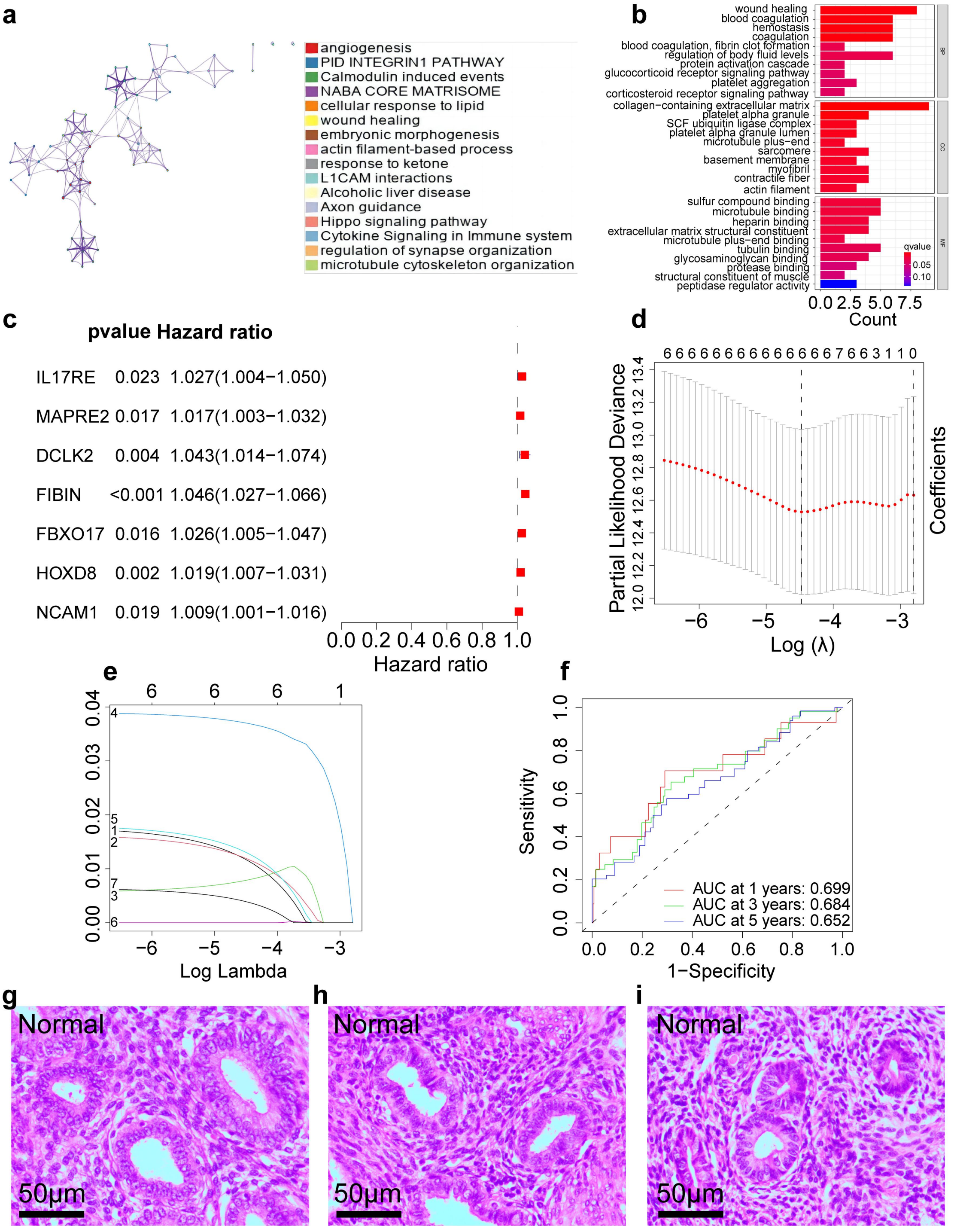

Fig. S11 (a) Metascape visualization of the interactome network formed by the 44 intersecting genes. (b) GO functional enrichment analysis of 44 intersecting genes, including cellular component (CC), molecular function (MF) and biological process

(BP). High expression, red. Low expression, blue. The length of the bar chart indicates the count of genes enriched. (c) Univariate Cox regression analysis was used to assess the genes that related to prognosis. (d) Feature selection was conducted using the LASSO regression model through 10-fold crossvalidation and lambda 1se. Coefficient distribution plots were generated for the log (lambda) sequence. (e) LASSO non-zero coefficient 6 significant genes in UCEC. (f) ROC curve analyses in predicting 1-, 3-, and 5-year overall survival (OS) in the TCGA-UCEC cohorts. (g-i) Hematoxylin and eosin (H&E) staining was performed to observe pathological changes in normal endometrial tissues (bar = 50 μm).

Fig. S12

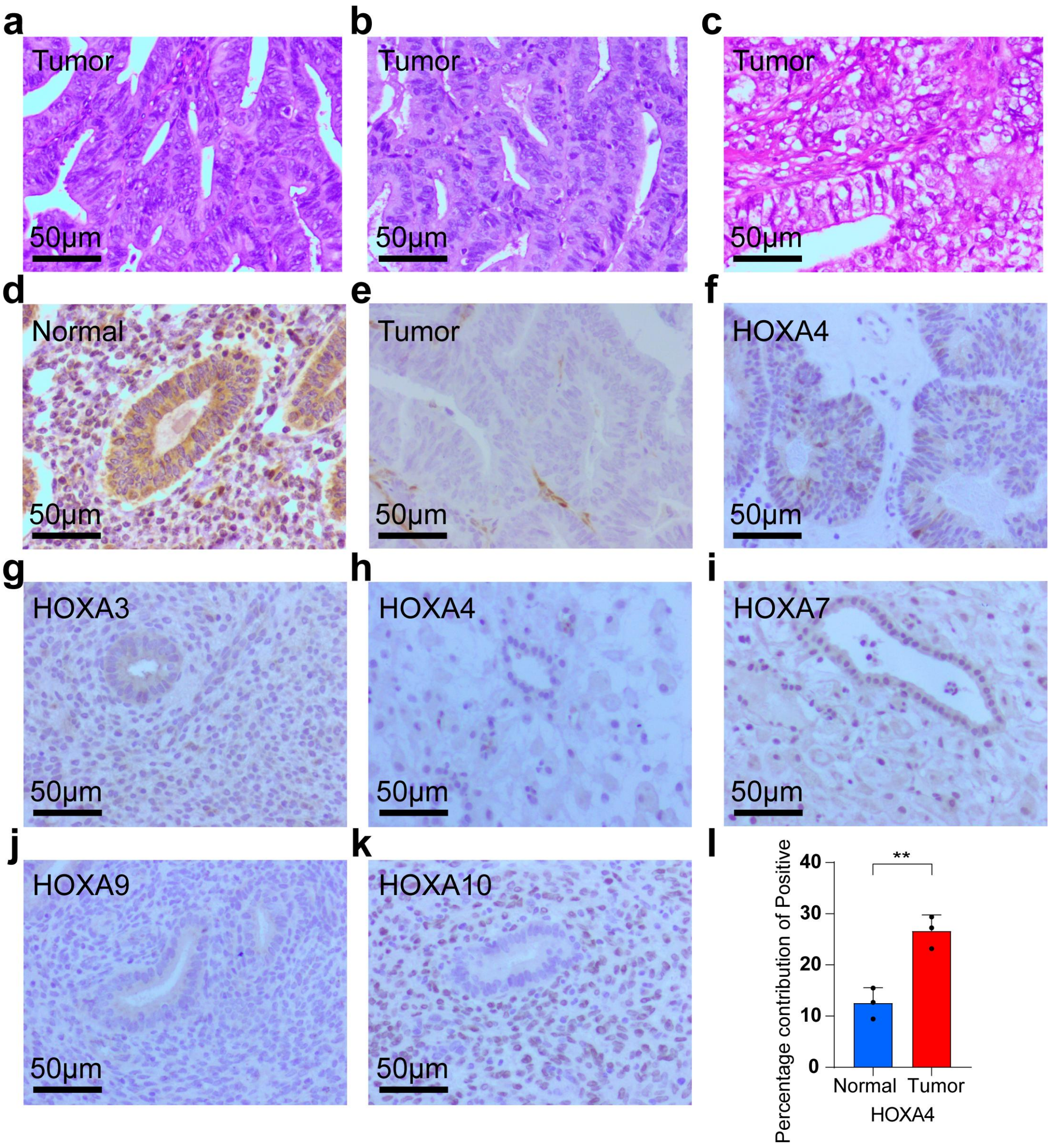

Fig. S12 (a-c) Hematoxylin and eosin (H&E) staining was performed to observe pathological changes tumor tissues (bar = 50 μm). (d-e) Differential immunohistochemical expression of PTEN between normal endometrial tissues and tumor tissues (d: normal; e: tumor) (bar = 50 μm). (f) Immunohistochemistry of the tumor tissue was performed against *HOX* gene. (g-k) Immunohistochemistry of the normal endometrial tissue was performed against *HOX* gene. (l) Percentage of

positive staining for *HOX* gene (control group: normal endometrial tissue; experimental group: endometrial tumor tissue. *, $P < 0.05$; **, $P < 0.01$; ***, $P < 0.001$).

Table. S1 Comparison of basic data from eligible selected endometrial cancer patients and controls. Including age, tumor size, histology, histological grading.

**Patient**		**Ca1**	**Ca2**	**Ca3**	**N1**	**N2**	**N3**
**Characteristic**	**Age**	54	60	49	52	55	50
	**Tumor size (cm)**	3.5*2.5*1.0	3.0*2.0*1.0	3.0*2.5*0.3	-	-	-
	**Histology**	Adenocarcinoma	Adenocarcinoma	Adenocarcinoma	-	-	-
	**Histological grading**	G1	G2	G1	-	-	-

a: Patients in the experimental group were annotated as Ga1, Ca2 and Ca3, respectively. Patients in the control group were labeled as N1, N2 and N3, respectively.

b: Histological grading is divided into four levels, G1, G2, G3 and G4.

Table. S2 Comparison of basic data from eligible selected endometrial cancer patients and controls. Including age, tumor size, histology, histological grading.

**Patient**		**Ca1**	**Ca2**	**Ca3**	**N1**	**N2**	**N3**
**Characteristic**	**Age**	58	57	46	58	58	50
	**Tumor size (cm)**	7.0*5.0*4.0	4.0*3.0*1.0	3.0*3.0*2.0	-	-	-
	**Histology**	Adenocarcinoma	Adenocarcinoma	Adenocarcinoma	-	-	-
	**Histological grading**	G2	G1	G1	-	-	-

a: Patients in the experimental group were annotated as Ga1, Ca2 and Ca3, respectively. Patients in the control group were labeled as N1, N2 and N3, respectively.

b: Histological grading is divided into four levels, G1, G2, G3 and G4.

Table. S3 Comparison of basic data from eligible selected endometrial cancer patients and controls. Including age, tumor size, histology, histological grading.

**Patient**		**Ca1**	**Ca2**	**Ca3**	**N1**	**N2**	**N3**
**Characteristic**	**Age**	63	54	46	61	50	55
	**Tumor size (cm)**	1.2*1.0*0.8	1.5*1.5*0.8	6.0*6.0*5.5	-	-	-
	**Histology**	Adenocarcinoma	Adenocarcinoma	Adenocarcinoma	-	-	-
	**Histological grading**	G1	G2	G2	-	-	-

a: Patients in the experimental group were annotated as Ga1, Ca2 and Ca3, respectively. Patients in the control group were labeled as N1, N2 and N3, respectively.

b: Histological grading is divided into four levels, G1, G2, G3 and G4.

Table. S4 Comparison of basic data from eligible selected endometrial cancer patients and controls. Including age, tumor size, histology, histological grading.

**Patient**		**Ca1**	**Ca2**	**Ca3**	**N1**	**N2**	**N3**
**Characteristic**	**Age**	60	62	50	60	61	50
	**Tumor size (cm)**	1.5*1.5*0.8	5.5*4.0*4.0	4.0*2.5*0.5	-	-	-
	**Histology**	Adenocarcinoma	Adenocarcinoma	Adenocarcinoma	-	-	-
	**Histological grading**	G2	G2	G1	-	-	-

a: Patients in the experimental group were annotated as Ga1, Ca2 and Ca3, respectively. Patients in the control group were labeled as N1, N2 and N3, respectively.

b: Histological grading is divided into four levels, G1, G2, G3 and G4.

Table. S5 Comparison of basic data from eligible selected endometrial cancer patients and controls. Including age, tumor size, histology, histological grading.

**Patient**		**Ca1**	**Ca2**	**Ca3**	**N1**	**N2**	**N3**
**Characteristic**	**Age**	53	59	54	58	53	55
	**Tumor size (cm)**	5.0*2.5*1.0	4.0*3.0*2.0	3.0*2.0*1.0	-	-	-
	**Histology**	Adenocarcinoma	Adenocarcinoma	Adenocarcinoma	-	-	-
	**Histological grading**	G2	G1	G2	-	-	-

a: Patients in the experimental group were annotated as Ga1, Ca2 and Ca3, respectively. Patients in the control group were labeled as N1, N2 and N3, respectively.

b: Histological grading is divided into four levels, G1, G2, G3 and G4.
